# Heart‐Specific Spinal and Vagal Afferents: Transcriptomic Signatures and Optogenetically Modulated Functional Coupling With Cardiomyocytes

**DOI:** 10.1002/cph4.70203

**Published:** 2026-06-30

**Authors:** T. Akgul Caglar, Y. E. Kazci, Z. B. Durdu, S. Sahoglu Goktas, S. Bay, E. Vatandaslar, M. U. Turhan, G. Ozturk, E. Cagavi

**Affiliations:** ^1^ Research Institute for Health Sciences and Technologies (SABITA), Istanbul Medipol University Istanbul Türkiye; ^2^ Neuroscience Program, Institute of Health Sciences Istanbul Medipol University Istanbul Türkiye; ^3^ Medical Biology and Genetics Program, Institute of Health Sciences Istanbul Medipol University Istanbul Türkiye; ^4^ Department of Cardiovascular Surgery, Haseki Training and Research Hospital University of Health Sciences Istanbul Türkiye; ^5^ Department of Physiology, School of Medicine Bolu Abant Izzet Baysal University Bolu Türkiye; ^6^ Department of Medical Biology, School of Medicine Istanbul Medipol University Istanbul Türkiye

**Keywords:** dorsal root and nodose ganglion, heart‐specific sensory neurons, neuro‐cardiac interactions, optogenetic modulation of neuro‐cardiac cultures, retrograde labeling, transcriptomic profile

## Abstract

Sensory neurons innervating the heart transmit chemical and mechanical cues to the central nervous system via the dorsal root ganglion (DRG) and nodose ganglion (NG). Despite their importance in cardiac pain and cardiovascular reflexes, the molecular and functional properties of heart‐specific sensory (HS) neurons remain elusive. Here, DRG^HS^ and NG^HS^ neurons innervating the heart were FACS purified using a retrograde labeling strategy with Di‐8‐ANEPPQ, then characterized molecularly by bulk RNA sequencing or evaluated functionally in cocultures with neonatal cardiomyocytes. DRG^HS^ and NG^HS^ neurons formed functional connections with neonatal cardiomyocytes as demonstrated by immunolabeling, electron microscopy, and optogenetic manipulation. Functionally coupled DRG^HS^ neurons exhibited enhanced spontaneous and evoked Ca^2+^ activity in response to optogenetic and chemical stimulation, indicating dynamic neuro‐cardiac communication. Global RNA sequencing analysis revealed that DRG^HS^ and NG^HS^ neurons exhibited distinct transcriptomic profiles, including enrichment of transcripts encoding ion channels and G protein‐coupled receptors, compared with their respective total populations. In DRG^HS^ tissue, these included *Scn10a*, *P2xr2*, and *Mrgprd*, whereas NG^HS^ neurons preferentially expressed *P2xr2*, *Ptgdr*, and *Cckar*, collectively supporting a combination of molecular signatures including nociceptors. Collectively, these findings define molecularly distinct sensory pathways connecting the heart and the sensory nervous system, providing mechanistic insights and highlighting potential targets for modulation of cardiovascular reflexes and hemostasis.

## Graphical abstract

Selective purification of heart‐specific spinal and vagal sensory neurons by retrograde labeling and FACS purification showed enhanced spontaneous, optogenetic, and chemically stimulated Ca^2+^ activity. Transcriptomic profiling identified distinct ion channel and GPCR signatures in heart‐specific sensory neurons. Molecular and functional profiles of cardiac innervating sensory neurons provided novel insights.

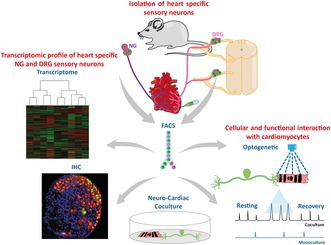

**Abbreviations**
Ca^2+^
: calcium
ChR2: chanallerhopsin‐2
Di‐8‐ANEPPQ: 1‐(3‐Trimethylammoniopropyl)‐4‐[−[2‐(di‐n‐octylamino)‐6naphthyl]vinyl]pyridinium dibromide
DiI: 1,1′‐Dioctadecyl‐3,3,3′,3′‐Tetramethylindocarbocyanine Perchlorate; DiIC18(3)
DRG: dorsal root ganglion
DRG^HS^
: heart‐specific dorsal root ganglion
DRG^T^
: total dorsal root ganglion
FACS: fluorescently activated cell sorting
GCaMP6s: genetically encoded Ca^2+^ biosensor
GCPR: G‐coupled protein receptor
Mrgprd: MAS related GPR family member D
NG: nodose ganglion
NG^HS^
: heart‐specific nodose ganglion
NG^T^
: total nodose ganglion
P2rx2: purinergic receptor P2X2
SCN10A: sodium voltage‐gated channel alpha subunit 10
TRP: transient receptor potential
TRPM8: transient receptor potential melastatin 8
TRPV1: transient receptor potential vanilloid 1
VSN: vagal sensory neurons

## 1 Introduction

The heart is innervated by sensory afferents that continuously transmit information about mechanical stretch, pressure, pain, and other sensory cues to the central nervous system (Armour 2010; van Weperen and Vaseghi 2023). Sensory input from the heart is conveyed through two anatomically and functionally distinct pathways: spinal and vagal nerves, in which neuronal bodies reside in the dorsal root ganglion (DRG) and nodose ganglion (NG), respectively. The sensory feedback plays a critical role in maintaining cardiac hemostasis. Any disturbance in the sensory circuit may contribute to arrhythmogenesis, autonomic imbalance, and maladaptive cardiac remodeling, particularly following ischemic injury (Armour 2010).

Retrograde labeling strategies have been widely used to identify innervated sensory neurons at end‐organs (Corbett et al. 1999; Guić et al. 2010; Hopkins and Armour 1989; Kosta et al. 2010; Krasteva et al. 2011; Wang and Miller 2016). Application of retrograde lipophilic dyes into the heart has revealed that cardiac afferents are distributed bilaterally across cervical and upper thoracic DRGs between C5 and T4 in addition to the vagal route in NG (Akgul Caglar, Durdu, et al. 2021; Guić et al. 2010; Hayakawa et al. 2011; Quigg 1991). Retrogradely labeled cardiac afferents were found to have a small cell diameter (< 20 μm) showing immunoreactivity for transient receptor vanilloid 1 (TRPV1) (Akgul Caglar, et al. 2021) and were responsive to ATP and adenosine (Benson et al. 1999), suggesting their nociceptor‐like identity. However, most investigations have been limited to tissue‐level analyses, leaving unresolved questions regarding the transcriptional diversity, ion channel repertoire, and signaling mechanisms that distinguish heart‐specific sensory neurons from the respective total sensory population.

Recent advances in transcriptomic profiling have greatly expanded our understanding of peripheral sensory neuron heterogeneity (Kupari et al. 2019; Zeisel et al. 2018). Both bulk (Chiu et al. 2014; Lopes et al. 2017; Thakur et al. 2014) and single‐cell RNA sequencing (Kupari et al. 2019; Usoskin et al. 2015; Zeisel et al. 2018) approaches have revealed molecular diversity within DRG and NG neurons. DRG neurons are classified into several subgroups, such as neurofilament, tyrosine hydroxylase, non‐peptidergic, or peptidergic groups, using their transcriptomic profile (Usoskin et al. 2015; Zeisel et al. 2018). Furthermore, purified nociceptive DRG neurons labeled with SCN11A marker were characterized using bulk RNA sequencing to highlight their pathways for pain sensation (Thakur et al. 2014). Similarly, transcriptomic analyses of NG neurons have identified 18 distinct subtypes characterized by ion channel and G‐protein coupled receptors (GPCR) profile, reflecting their functional similarities to low‐threshold mechanoreceptors or nociceptive DRG neurons (Kupari et al. 2019). Furthermore, Zhao et al. reported retrograde labeling of seven visceral organs, including the heart and lung, and evaluated the projection of vagal sensory neurons (VSN) in PHOX2b+ NG and PRDM12+ jugular ganglia (Zhao et al. 2022). Their data revealed that the gene expression profile of molecularly distinct and organ‐specific populations of VSNs stemmed mainly from the NG but not the jugular ganglia (Zhao et al. 2022). However, the functional relevance of the molecular identity of heart‐specific NG neurons was not explored in detail. Importantly, this study provides the first comprehensive global transcriptomic profile of the heart‐specific DRG neurons, a population that remains poorly characterized, and the fundamental molecular and sensory modality differences between vagal and spinal afferent pathways to the heart.

In addition to the molecular profiling, the functional coupling between cardiac sensory neurons and cardiomyocytes remains poorly understood. In vitro coculture systems have demonstrated bidirectional communication between cardiomyocytes and autonomic neurons, whereby stimulation of sympathetic or parasympathetic neurons modulates the beating rate of heart muscle cells (Takeuchi et al. 2011; Hick et al. 2013; Oh et al. 2016). In contrast, analyses of cardiac sensory neurons are limited (Hoebart et al. 2023, 2024). Understanding how cardiomyocyte activity influences sensory neuron excitability is critical for elucidating mechanisms of cardiac pain, reflex control, and signaling in cardiac disorders.

In the present study, we combined retrograde labeling, fluorescence‐activated cell sorting, RNA sequencing, and Ca^2+^ imaging, combined with optogenetic/chemical stimulation, to characterize spinal and vagal sensory neurons innervating the heart. Heart‐specific DRG (DRG^HS^) and NG (NG^HS^) sensory neurons were selectively labeled using Di‐8‐ANEPPQ and purified by fluorescent‐activated cell sorting (FACS) for functional or transcriptomic analysis. In cocultures, DRG^HS^ neurons were functionally coupled with cardiomyocytes, with enhanced spontaneous Ca^2+^ activity and dynamics with increased amplitude and interval in response to optogenetic and chemical stimulation. Moreover, transcriptomic profiling identified distinct ion channels and GPCR signatures in DRG^HS^ and NG^HS^. Transcriptomic and functional properties of different heart‐specific neural populations will allow for monitoring and understanding the control of heart function, shedding light on the previously unknown cellular and molecular basis of the interaction between the heart and the sensory nervous system.

## 2 Materials and Methods

### 2.1 Experimental Animals

All animal experiments were approved by the Animal Research Ethics Committee of Istanbul Medipol University under approval number 38328770‐30 and were conducted in accordance with the guidelines of the National Agriculture and Forest Ministry. All animals were housed under standard laboratory conditions with a 12 h light/dark cycle and had ad libitum access to food and water. Experiments were conducted on wild‐type female BALB/c mice aged 8–12 weeks (*n* = 24) for in vivo labeling, FACS‐sorting, and further evaluation of the heart‐specific DRG and NG neurons. Mrgprd‐Cre::tdTomato transgenic mice (JAX stock #031286, *n* = 2) were used to briefly validate transcriptomic findings. The genotype of each mouse was validated using the protocols provided by the supplier (JAX lab). For the coculture assay, neonatal cardiomyocytes were isolated from P0–3 litter mice (*n* = 9).

### 2.2 In Vivo Labeling of Heart‐Specific Sensory Neurons

Cardiac sensory neurons were labeled in vivo as previously described (Akgul Caglar, Durdu, et al. 2021). Briefly, the animals were anesthetized with intraperitoneal injection of ketamine (50 mg/kg, Pfizer) and xylazine (5 mg/kg, Bayer). Depth of anesthesia was assessed by monitoring pedal withdrawal reflex and eye movement; additional doses were administered if required. Following induction of anesthesia, mice were endotracheally intubated using an intubation cannula with a Y adapter (1.2 mm OD, 27 mm length; 732844, Harvard Apparatus) and connected to a small‐animal ventilator (Harvard Apparatus Minivent Type 845). Animals were placed on a heating pad (WPI) to maintain body temperature, and all surgical procedures were performed under a stereomicroscope (Discovery 8, Zeiss). A thoracotomy was performed above the xiphoid process, and the third and fourth intercostal muscles were gently separated to expose the heart. After visualizing the cardiac apex, the pericardium layer was carefully removed, and 1 μL of the retrograde tracer Di‐8‐ANEPPQ (10 mg/mL; 61,014, Biotium) was injected into the apex using a Hamilton syringe (701N, 30G). For sham control animals, 1 μL DMSO (vehicle for Di‐8‐ANEPPQ) was injected into the same region to enable comparison of gene expression profile between the Di‐8‐ANEPPQ labeled cardiac afferents and the total sensory population. Following the dye or vehicle injection, gentle negative pressure was applied, and the intercostal muscles and skin were closed using 6–0 silk sutures (S2165, Dogsan).

### 2.3 Sensory Neurons Isolation and Culture

Seven days after the Di‐8‐ANEPPQ and vehicle injection, mice were euthanized by CO_2_ asphyxiation. First, bilateral NGs and DRGs from cervical to thoracic spinal segments were collected as previously described (Cengiz et al. 2012). Dissected tissues were immediately transferred to ice‐cold RPMI 1640 medium (R0883, Gibco). After trimming nerve fibers, ganglia were enzymatically dissociated in neural medium containing 100 U/mL collagenase from *Clostridium histolyticum* , type XI (C7657, Sigma) supplemented with 2% B27 (17504‐044, Gibco), 2 mM Glutamax‐I (35050‐61, Gibco), 100 U penicillin/Streptomycin (15140‐122, Gibco), and 100 mg streptomycin (15140‐122, Gibco) in Neural Basal Medium (NBA, 10888‐022, Gibco) at 37°C in a 5% CO_2_ incubator for 40 min. Following collagenase digestion, tissues were washed with Hank's Balanced Salt Solution (H9269, Sigma) and further incubated in 1 mg/mL trypsin (25300‐054, Gibco) for 15 min at 37°C and 5% CO_2_. DNAse (50 lg/mL; D4513, Sigma) was then added, and the tissues were gently triturated to obtain a single‐cell suspension. After centrifugation at 120 g for 3 min, the pellet was dissolved in Neural Basal Medium supplemented with 10% fetal calf serum (10270‐106, Gibco) and 700 lg/mL trypsin inhibitor (T6522, Sigma). The cell suspensions from Di‐8‐ANEPPQ labeled DRGs and NGs were centrifuged at 120 g for 3 min and transferred directly for FACS‐sorting. In parallel, the cell suspension from unlabelled‐total DRG SHAM control group was applied to 10%, 35%, and 60% percoll gradient layers (P4937, Sigma) and was centrifuged at 200 g for 20 min. The percoll gradient clean‐up protocol has been well‐established in the literature for the purification of primary sensory neurons by eliminating non‐neuronal components (satellite glial cells, erythrocytes, myelin fragments, cellular debris etc.), without introducing a bias for specific cell size (Goldenberg and De Boni 1983; Walker et al. 2012; Lee and Levine 2015; Kaval Oğuz and Öztürk 2019; Aydın et al. 2023). The enriched neuron soma was isolated from the 35% percoll layer following centrifugation at 120 g for 3 min. The pellet was resuspended in a neural medium for further analysis.

### 2.4 Neonatal Cardiomyocyte Isolation and Culture

For neonatal cardiomyocyte culture, P0‐3 litter mice were used as previously described (Lee et al. 2012). The dissected hearts were put on ice‐cold Hank's Balanced Salt Solution (14170‐088, Gibco) to remove the blood, atria, and connective tissue. Then, the apex of the heart was slightly cut and put on 0.05% Trypsin EDTA (25300‐054, Gibco) at 4°C overnight. The next day, the enzyme solution was partially replaced with DMEM (41966‐029, Gibco) containing 25% M‐199 (31150‐022, Gibco), 1% HEPES (1×, H0887, Sigma), 1% Glutamax (35050‐61, Gibco), 1% P/S (15140‐122, Gibco), and the tissues were incubated at 37°C for 1–2 min. Following the incubation, the media was refreshed with DMEM (41966‐029, Gibco) containing 20% M‐199 (31150‐022, Gibco), 1% HEPES (1×, H0887, Sigma), 1% Glutamax (35050‐61, Gibco), 1% penicillin/streptomycin (15140‐122, Gibco), 10% FBS (10270‐106, Gibco), 5% Horse serum (16050‐130, Gibco) to inhibit the enzymatic activities. Then, the tissues were digested in 70% collagenase type II (17101‐015, Gibco) at 37°C for 40 min. The resulting cell suspension was collected, mixed with Cardiomyocyte Medium (1% Glutamax, 1% P/S, 1% MEM NEAA (11140‐050, Gibco), 1% Sodium Pyruvate (11360‐070, Sigma), 3% FBS, and 1.5% Horse Serum in DMEM), and centrifuged at 600‐800 RPM for 3–5 min. To reduce fibroblast contamination, cells were pre‐plated on 1% gelatin (G9391, Sigma)‐coated dishes for 1–2 h. The cardiomyocyte‐rich supernatant was collected, centrifuged (1000 RPM, 5 min), and resuspended in the Cardiomyocyte medium supplemented with 2% B27 (17504‐044, Gibco). The neonatal cardiomyocytes were seeded onto aprecoated poly‐l‐lysine (P6282, Sigma) with laminin (1 mg/mL; L2020, Sigma) and fibronectin (1 mg/mL; F1141, Sigma) coated petri dish for monoculture or cocultures. Ara‐C (C1768, Sigma) at 5 μM concentration was applied to all petri dishes for 12 h to minimize fibroblast and glia contamination.

### 2.5 Fluorescent‐Activated Cell Sorting (FACS)

Prior to purifying Di‐8‐ANEPPQ labeled neurons, we first evaluated cell viability of sensory neurons following enzymatic dissociation and/or percoll gradient. The cell suspension was incubated with 1 μg/mL Propidium Iodide (PI; P4170, Sigma) and 1 mg/mL Hoescht 33342 (14533, Sigma) to mark necrotic cells and nuclei, respectively. Following 30 min at 37°C incubation, the suspension was centrifuged at 120 g for 3 min and resuspended with Ca^2+^‐ and Mg^2+^‐free PBS (14190‐094, Gibco) supplemented with 1% FBS (10270‐106, Gibco) and 1% DNAse (D4513, Sigma). After filtration through a 100 μm cell strainer (352360, Falcon), the samples were analyzed using BD FACS Influx. Single Hoechst‐positive and PI‐negative (viable) neurons were identified and gated using BD FACS Software based on forward/side scatter, trigger pulse width, 355 (460/50), and 561 (593/40) filters.

DRG and NG neurons labeled with Di‐8‐ANEPPQ were purified using a FACS‐sorter (BD Biosciences). The enzymatically dissociated neurons (as described above) were resuspended with PBS without CaCl_2_, and MgCl_2_ (14190‐094, Gibco) supplemented with 1% FBS (10270‐106, Gibco) and 1% DNAse (D4513, Sigma), and filtered through a 100 μm cell strainer (352360, Falcon). Di‐8‐ANEPPQ labeled neurons were analyzed and sorted on a BD FACS Influx cell sorter set up with a 488 laser, 100 μm nozzle at 17 PSI. Di‐8‐ANEPPQ positive neurons at the single‐cell level were identified and gated by BD FACS Software using forward side scattered, trigger pulse with a 488 (670/30) filter. Di‐8‐ANEPPQ positive cells were sorted directly into neural basal medium supplemented with 2% B27, 1% Glutamax‐I (35050‐61, Gibco), 1% penicillin/streptomycin (15140‐122, Gibco), and 25 ng/mL NGF (N6009, Sigma). For live cell imaging or immunocytochemistry, the sorted Di‐8‐ANEPPQ labeled cells were seeded either as monocultures or co‐cultures with cardiomyocytes. For RNA sequencing, the sorted Di‐8‐ANEPPQ cells were centrifuged at 120 g for 3 min, and the pellet was lysed for RNA isolation.

### 2.6 Optogenetic Analysis

For optogenetic stimulation, the cardiomyocytes were transduced with AAV‐CAG‐ChR2‐H134R‐tdTomato (#28017, Addgene) prior to co‐culture with FACS‐purified neurons (Hunnicutt et al. 2014). To visualize Ca^2+^ dynamics, monocultures or cocultures were transduced with AAV‐EF1a‐GCaMP6s‐WPRE‐pGHpA (#67526, Addgene) (Chen et al. 2013; Wertz et al. 2015). AAV particles (MOI above 10^11^) were resuspended in Cardiomyocyte Medium without penicillin/streptomycin and incubated for 24 h, and then replaced with fresh medium. At 4 or 5 days post‐transduction, the reporter proteins (GFP for GCaMP6s and tdTomato for CHR2) were verified using confocal microscopy. The optogenetics stimulation was performed under LSM 780 confocal microscopy equipped with Plan‐Apochromat 10×/0.45 M27 objective. The GCaMP6s and CHR2 fluorescent reporters were detected using a 488 nm and a 561 nm excitation and 493–556 nm and 575–691 nm emission filters, respectively. Neonatal cardiomyocytes with CHR2 were selected using the research of interest (ROI) mode, and blue light (488 nm) was delivered in bleaching mode at 0.2 or 1 Hz for 100 s to the selected cardiomyocyte. To assess functional coupling, the Ca^2+^ transients in sensory neurons axonally connected to the optogenetically stimulated cardiomyocyte were continuously recorded during spontaneous baseline, optogenetic stimulation, and at recovery phases. All images were analyzed using the Zen (Zeiss) or Image‐J (https://imagej.net; version.1.52i) program. Basal fluorescence intensities were analyzed using a one‐phase decay function in GraphPad Prism software to normalize GCaMP6s signals to 100%. Raw fluorescence intensity was denoted as *F*, and normalized fluorescence intensity is denoted as *F*_*0*_. The [(F/F0)‐1] *100 formula was used to obtain normalized signal curves. Maximal amplitude was defined as the peak value of the normalized signal. The event duration was measured between the onset and termination of maximal amplitude. The number of events was quantified by counting individual Ca^2+^ peaks for spontaneous, chemical, or optogenetic stimulation recordings.

### 2.7 Real‐Time Ca^2+^ Imaging

Real time Ca^2+^ imaging was performed using GCaMP6s vectors by excitation with a 488 nm laser, and emitted fluorescence was collected at 509 nm. Imaging experiments were performed using Spinning disk microscopy equipped with LD A‐plan 20×/0.3 dry objective, Yokogawa CSU‐X1 (Tokyo, Japan) confocal scanner unit, and QuantEM:512SC (Teledyne Photometrics, AZ, USA). Each recording was for 60–120 s from cultures up to 25 days. Neurons were imaged before and after chemical stimulation in the absence or presence of 100 nm Isoproterenol hydrochloride (35100, USP).

### 2.8 Immunostaining

For immunohistochemical characterization of Di‐8‐ANEPPQ labeled DRGs and NGs, mice were euthanized 7 days following Di‐8‐ANEPPQ injection. NG and DRG tissues were embedded in OCT (14020108926, Leica) and cryo‐sectioned at 10 μm thick. Due to PFA‐induced fluorescence quenching of Di‐8‐ANEPPQ (Akgul Caglar, Durdu, et al. 2021), Di‐8‐ANEPPQ images were acquired prior to fixation and subsequently merged with the corresponding immunolabeled images at both tissue and single‐cell levels. XY alignment was achieved through a multi‐step spatial registration process in ImageJ, utilizing bright‐field (BF) images as a structural reference. BF and fluorescent images were acquired at both tissue and single‐cell levels before and after fixation. The BF images served as a spatial guide to align the two sets of coordinates via translation and rotation. Once registered, the pre‐fixed Di‐8‐ANEPPQ fluorescent signal was merged with corresponding post‐fixation immunolabeled channels, using the Merge Channels module. This process ensured that fluorescently labeled cells matched the molecular markers identified after processing. Next, sections were fixed in 4% PFA (158127, Sigma) for 15 min and treated in blocking buffer (3% bovine serum albumin (BSA; A9418, Sigma), 0.01% sodium azide (S8032, Sigma), 5% serum (16210‐064, Gibco), and 0.1% Triton X‐100 (X100, Sigma) in PBS) for 1 h. In parallel, mono‐ and cocultures were immunolabeled using the same protocol described for tissue sections to measure soma size, neurite diameter, and neurite number. Tissue or cell culture samples were incubated overnight at 4°C with primary antibodies against selected markers: rabbit anti‐P2XR2 (1:200; APR‐003, Alomone Labs), rabbit anti‐SCN10a (1:200; ASC‐016, Alomone Labs), rabbit anti‐Tuj1 (1:1000; ab18207, Abcam), mouse anti‐TRPV1 (1:200; ab203103, Abcam), or mouse anti‐cTnT (1:500; MS‐295‐P, Thermo). Following primary antibody incubation, sections were treated with Alexa Fluor 488 anti‐rabbit IgG (1:500; 4408S, CST) and/or Alexa Fluor 647 anti‐mouse IgG (1:500; 4414S, CST) for 1 h at RT, followed by nuclear staining with DAPI (1 μg/mL; D9542, Sigma) for 5 min at RT. All fluorescence images from tissue sections and cultured cells were acquired using an LSM 780 confocal microscope (Zeiss).

### 2.9 RNA Isolation and Library Construction

RNA was isolated from the following samples using the RNeasy micro kit (74034, Qiagen). RNA concentration from FACS‐purified Di‐8‐ANEPPQ labeled DRG (DRG^HS^; *n* = 3 biological replicates, 3–4 animals pooled per replicate), purified Di‐8‐ANEPPQ labeled NG (NG^HS^; *n* = 2 biological replicates, 4 animals pooled per replicate), SHAM total DRG population (DRG^T^; *n* = 2 biological replicates from 2 animals), and SHAM total NG population (NG^T^; *n* = 2 biological replicates from 2 animals) was determined using a Qubit RNA Quantification kit (Q32852, Invitrogen) and Qubit fluorometer (Q33216, Thermo Scientific). For Global RNA sequencing analysis, 500 ng/μL and 200 ng/μL of RNA were used for DRG and NG samples, respectively. RNA integrity was assessed using the Agilent 2100 Bioanalyzer, in which samples with RNA integrity number (RQN) as shown in Table S10 were included in the sequencing reaction. RNA libraries were prepared using the TruSeq Total RNA Sample Preparation Kit v2 (15058251, Illumina) and sequenced using paired‐end reads (2 × 75 bp) on the Illumina NextSeq 500 platform (Illumina) at Istanbul Medipol University Genomic Center.

### 2.10 qRT‐PCR

Complementary DNA was synthesized from 200 ng RNA using the iScript Advanced cDNA Synthesis Kit (170‐8891, Bio‐Rad). qRT‐PCR was performed in triplicate using iTaq Universal SYBR Green Supermix (170‐8882, Bio‐Rad) on the CFX Bio‐Rad real‐time PCR system. Primer sequences are provided in Table S11. To ensure the molecular purity of the FACS‐isolated neuronal populations from DRG and NG, we employed a dual‐normalization strategy. First, target gene expression was normalized to the housekeeping gene *Gapdh* using the ΔCt method to account for variations in RNA input. To eliminate potential bias from trace cardiac mRNA in our FACS‐isolated samples, target gene expression was further normalized to *Myh6* as a lineage‐specific correction factor, ensuring the observed results were strictly neuronal in origin. This normalization served as a critical control for variations in cellular composition across samples; in the context of cardiac‐innervating neurons, *Myh6* serves as a high‐fidelity indicator of sample homogeneity. Final comparative analyses between groups were performed using the ΔΔCt method, with results expressed as relative fold change. This approach is consistent with previous studies suggesting that standard housekeeping genes may not provide sufficient normalization across varying biological contexts and that strategies should be tailored to specific sample composition (Suzuki et al. 2000; Vandesompele et al. 2002; Bustin et al. 2009).

### 2.11 Bioinformatics Analysis

On average, approximately 40 million reads were obtained per sample. Read quality was assessed using FastQC v0.11.6 (Anders 2010), which indicated that the proportion of low‐quality bases was negligible. Reads were not trimmed based on quality, as previous studies have shown that user‐defined trimming parameters can introduce bias and negatively affect downstream analyses (Williams et al. 2016). Transcript‐level quantification was performed using Salmon v0.8.2 (Patro et al. 2017) with the Ensembl v88 release of the mouse transcriptome (mm10). Gene‐level abundance estimates were generated using the Tximport package in R (Soneson et al. 2015). All downstream analyses were conducted in R (Huber et al. 2015).

Differentially expressed genes were identified using the DESeq2 package (Love et al. 2014). Genes with an absolute log_2_ (fold change) > 0.5 and multiple‐test adjusted *p* value < 0.05 were considered statistically significant (|log2FC| > 0.5, *p*adj < 0.05) (Schurch et al. 2016). Additionally, genes with an absolute log_2_(fold change) > 1.5 were quantified separately for emphasis (Schurch et al. 2016). Regularized log‐transformed counts generated by DESeq2 were used for principal component analysis (PCA) and hierarchical clustering based on Euclidean distance. PCA was generated using the 500 most variable genes across all samples (Love et al. 2014). Data visualization and statistical plots were generated using the ggplot2 and gplots R packages (Warnes et al. 2025). Functional enrichment analyses of differentially expressed genes were performed using the GOseq package (Young et al. 2010) to identify overrepresented Gene Ontology (GO) biological processes. Enriched pathways were further investigated using annotation data from the KEGG (Ogata et al. 1999) and Reactome databases (Gillespie et al. 2022). All raw and processed RNA‐seq data have been deposited in the Gene Expression Omnibus (GEO) under accession number GSE211622.

### 2.12 Statistical Analysis

All imaging data were analyzed using GraphPad Prism version 9 (GraphPad Software, San Diego, CA, USA). Each experiment was repeated a minimum of three times. Results were given as N/n, where N represents the number of independent experiments and *n* denotes the number of the cells analyzed. Data were presented as mean ± SD. Statistical significance between two groups was assessed using an unpaired Student's *t*‐test. For drug or optogenetic stimulation experiments, paired *t*‐test were applied to pre–post comparisons within the same cells. One‐way ANOVA with Tukey post hoc test was used for multiple‐group comparisons. The amplitude, the number of events, and the duration of spontaneous or pre‐post stimulation Ca^2+^ signals were analyzed using Pearson's correlation test. Correlations between RNA‐seq (FPKM) and qPCR (ΔΔCt) data were determined using Pearson's correlation coefficients in R. A *p*‐value < 0.05 was considered statistically significant.

## 3 Results

### 3.1 Sorting of Retrogradely Labeled Heart‐Specific Sensory Neurons and Morphological Characterization

**FIGURE 1:**
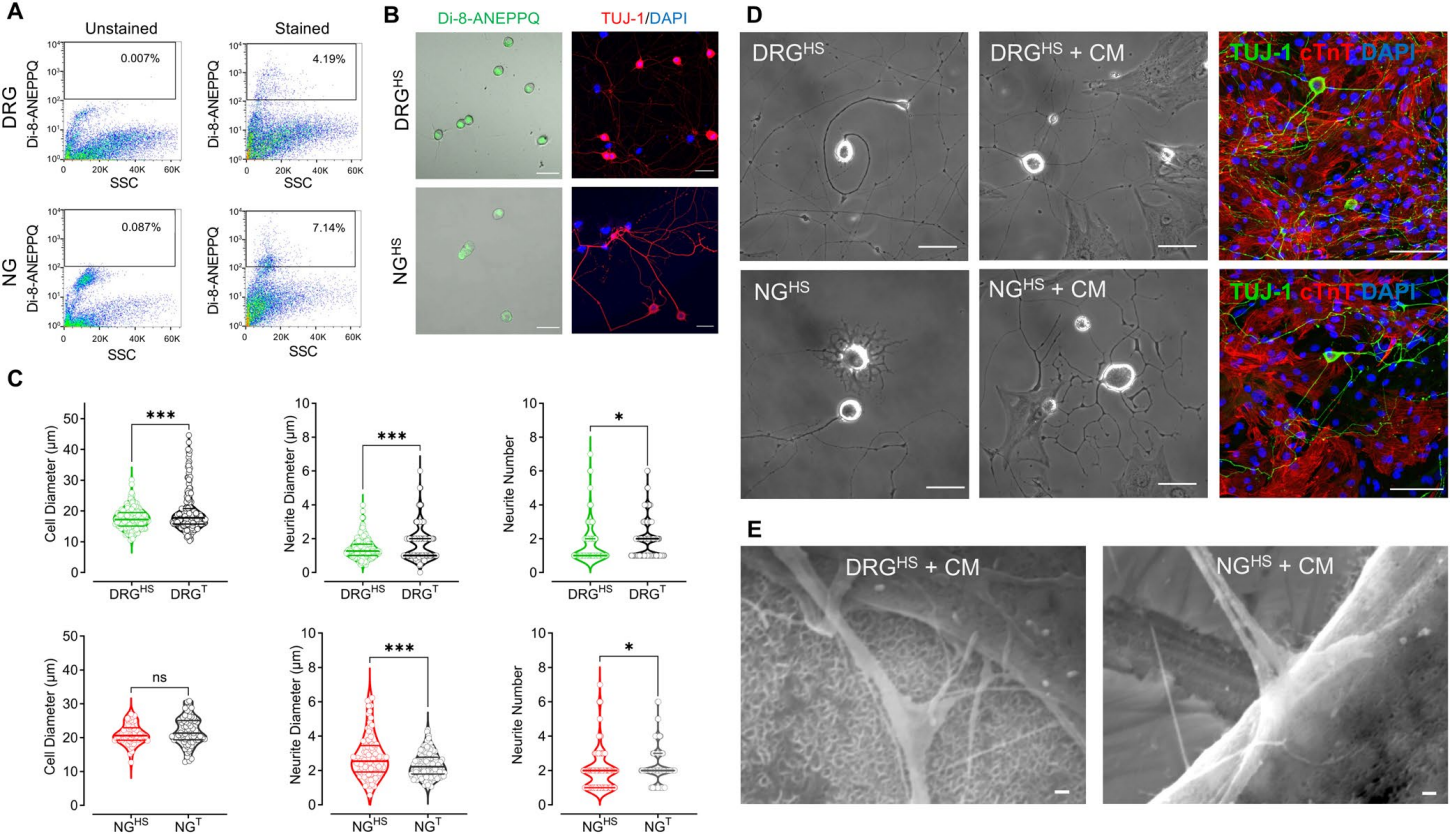
Morphological characterization of FACS‐sorted heart‐specific sensory neurons and cocultures with neonatal cardiomyocytes. (A) Representative FACS plot illustrating the gating and sorting strategy of DRG^HS^ and NG^HS^ sensory neurons that were retrogradely labeled with Di‐8‐ANEPPQ compared to unstained total (DRG^T^ and NG^T^) sensory populations. (B) Representative bright‐field and fluorescence images of Di‐8‐ANEPPQ‐labeled neurons imaged following FACS sorting and after 3 days in culture, immunolabeled for the neuronal marker TUJ‐1 (red) and nuclear marker DAPI (blue). Scale bar: 50 μm. (C) The upper panel illustrated cell diameter of DRG^HS^ (*n* = 202 cells), DRG^T^ (*n* = 200); neurite diameter of DRG^HS^ (*n* = 254), DRG^T^ (*n* = 310); and neurite number of DRG^HS^ (*n* = 150) and DRG^T^ (*n* = 158). The lower panel represented the cell diameter of NG^HS^ (*n* = 53), NG^T^ (*n* = 67); neurite diameter of NG^HS^ (*n* = 93), NG^T^ (*n* = 115), and neurite number of NG^HS^ (*n* = 150) and NG^T^ (*n* = 158). Values denoted mean ± S.D., *p < 0.05, ***p < 0.01, ns: non‐significant by Student's *t*‐test. Scale bar: 50 μm. (D) Representative bright field images of DRG^HS^ and NG^HS^ in monocultures or cocultures with neonatal CM. Immunostaining of cocultures with TUJ‐1, cardiac Troponin T (cTnT), and DAPI was pseudo‐colored with green, red, and blue, respectively. Scale bar: 50 μm. (E) High‐resolution scanning electron microscopy (SEM) images demonstrating the physical interaction between DRG^HS^ or NG^HS^ and neonatal CMs. Sensory neurons were branched and formed extensions with multiple point contacts with the underlying cardiomyocyte sarcolemma. Scale bars: 200 nm. DRG^HS^: Heart‐specific DRG sensory neurons, NG^HS^: Heart‐specific sensory neurons, CM: Cardiomyocytes.

To obtain an enriched population of heart‐specific sensory neurons in DRG and NG, the Di‐8‐ANEPPQ retrograde label was applied to the apex of the heart as we described previously (Akgul Caglar, et al. 2021). Following dissociation of DRG and NG tissues, the viability was determined to be approximately 93.4% and 86.6%, respectively, by flow cytometry analysis (Figure S1A). Di‐8‐ANEPPQ labeled heart innervating sensory neurons from DRGs of the C‐T segments, and bilateral NG tissues were FACS sorted, yielding 4.19% and 7.14%, respectively, of all viable cells (Figure 1A, Figure S1B). Following sorting, the Di‐8‐ANEPPQ‐labeled heart‐specific DRG^HS^ and NG^HS^ neurons were cultured to verify their fluorescence and their neurite extension for the subsequent days, exhibiting the pan‐neural marker TUJ‐1 immunolabeling (Figure 1B, Figure S1C). This data demonstrated that the cardiac innervating DRG^HS^ and NG^HS^ neurons were enriched by the FACS sorting strategy following the retrograde labeling with Di‐8‐ANEPPQ fluorescent dye.

The cardiac innervating DRG^HS^ and NG^HS^ neurons were characterized morphologically by soma diameter, neurite diameter, and the number of neurites of TUJ‐1 immunolabeled sensory neurons (Figure 1C). The DRG^HS^ neurons had an average of 17.55 ± 3.18 in soma size that was smaller than the cell diameter of the total sensory neuron population (19.48 ± 6.38, DRG^T^, *p* = 0.0001) (Table S12). On the other hand, the cell diameter of NG^HS^ was measured as 20.98 ± 2.99 μm, similar to the total NG population (21.92 ± 4.33 μm, NG^T^; *p* = 0.1786). Moreover, the average diameter of the neurites of DRG^HS^ neurons was measured to be 1.4 ± 0.58 μm, that was significantly thinner than the DRG^T^ neurons (1.63 ± 0.97 μm; *p* = 0.0003). In the NG^HS^ neuron culture, the neurite diameter of 2.82 ± 1.25 μm was determined to be thicker than the NG^T^ neurons (2.31 ± 0.70 μm; *p* = 0.0003). Moreover, cardiac innervating spinal (1.63 ± 1.07) and vagal sensory neurons (1.94 ± 1.09) extended less neurites than total afferent population (1.92 ± 1.1 for DRG^T^; 2.23 ± 1.04 for NG^T^; *p* = 0.019 and *p* = 0.017, respectively) (Figure 1C, Table S12). These data indicated distinct morphological characteristics between cardiac innervating sensory neurons from the total afferent population under these experimental conditions.

### 3.2 The Interaction of Heart‐Specific Sensory Neurons and Neonatal Cardiomyocytes In Vitro

Next, we cultured the sorted NG^HS^ or DRG^HS^ sensory neurons as a monoculture or co‐culture with neonatal primary cardiomyocytes to investigate their interaction in vitro. The sorted DRG^HS^ and NG^HS^ extended neurites and formed physical connections with each other and cardiac muscle cells immunolabeled with pan‐cardiac cTnT marker protein (Figure 1D). Further analysis by scanning electron microscopy revealed physical interactions between NG^HS^ or DRG^HS^ sensory neurons and cardiomyocytes (Figure 1E).

**FIGURE 2:**
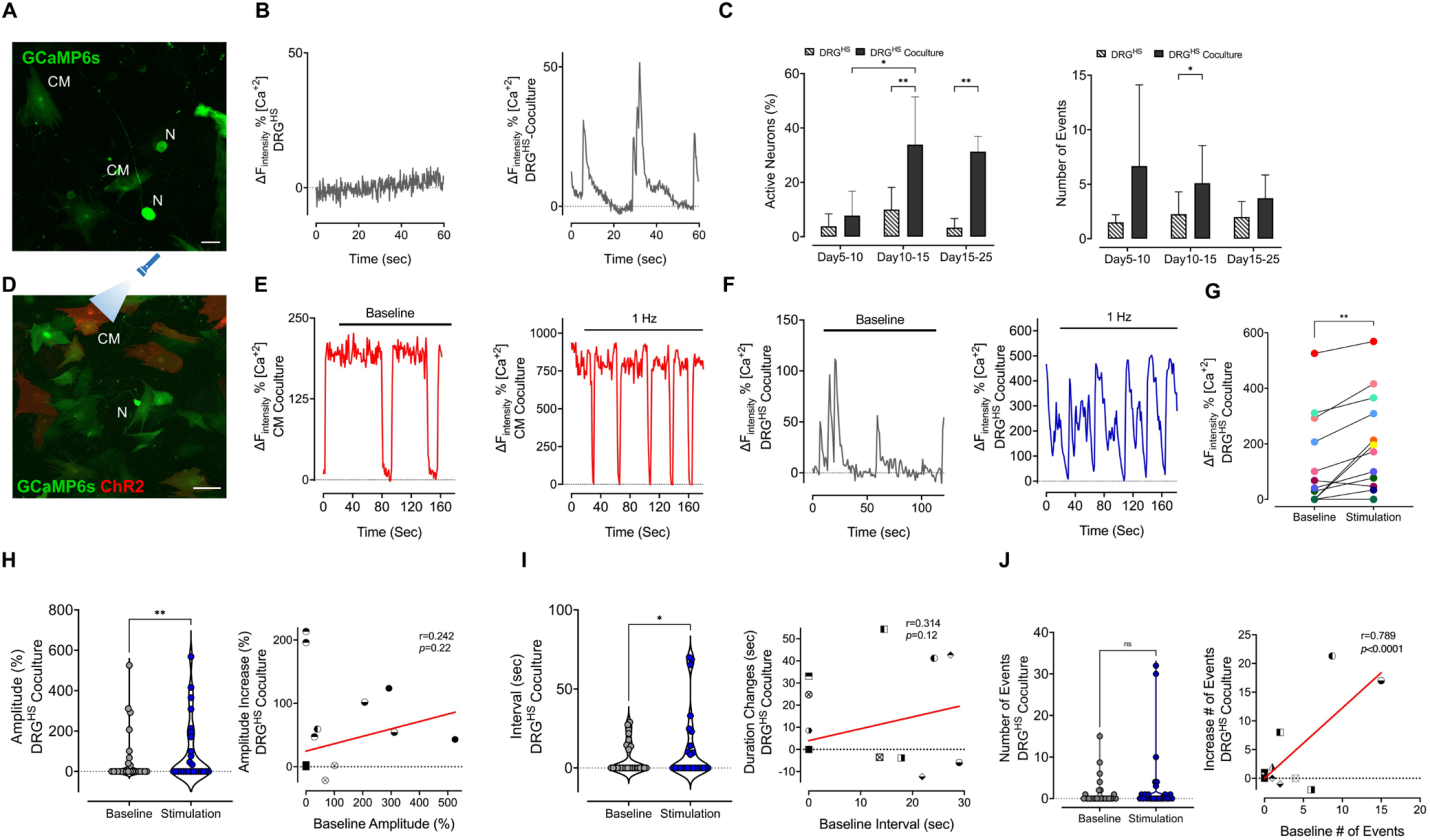
Functional characterization of DRG^HS^ neuron and cardiomyocyte cocultures at baseline and following optogenetic stimulation. (A) Representative image of GCaMP6s‐expressing (green) DRG^HS^ and CM displaying. Scale bar: 50 μm (B) spontaneous activity in mono and cocultures. (C) Quantitative comparison of DRG^HS^ Ca^2+^ transient signals across 5–25 days of mono and cocultures (*N* = 4 experimental replicates, *n* = 441 cells analyzed). (D) Representative image of cardiomyocytes expressing ChR2 (red) and/or GCaMP6s (green) together with DRG^HS^ neurons expressing GCaMP6s in coculture. Scale bar: 50 μm. (E) Representative Ca^2+^ transient signals of CM at baseline and with optogenetic stimulation at 1 Hz. (F) The subsequent response of DRG^HS^ following CM activity in (E). (G) Collective neuronal activity recorded in (F) through fluorescent Ca^2+^ changes at baseline and with optogenetic stimulation of CM. Violin plots and correlation analyses showing changes in amplitude (H), interval (I), and event frequency (J) in DRG^HS^ neurons following CM stimulation (*N* = 3, *n* = 26, *p < 0.05, **p < 0.01 by paired *t*‐test).CM: Cardiomyocytes, N: Neuron, DRG^HS^: Heart‐specific DRG sensory neurons.

To investigate the interaction between the cardiac innervating neurons and neonatal cardiomyocytes regarding functional Ca^2+^ coupling and activity, the sorted DRG^HS^ neurons, either cultured alone or with cardiomyocytes, were transfected with the Ca^2+^ biosensor GCaMP6s coding virus (Chen et al. 2013). Following transduction, both DRG^HS^ neurons and cardiac muscle cells displayed the GFP expression associated with GCaMP6s stably detected with confocal microscopy (Figure 2A). Spontaneous Ca^2+^ transients measured in mono and co‐cultures (Figure 2B) demonstrated a peak in % of active neurons at 10–15 days in DRG^HS^ cocultures reaching up to 33.89% ± 17.56, significantly higher than DRG^HS^ monocultures (10% ± 8.16, *p* = 0.004) (Figure 2C). Although the % of active neurons remained consistently higher in later days in cocultures, the number of DRG^HS^ neurons firing on days 10–15 was measured to be 5.1 ± 3.45 in cocultures, which was higher than monocultures (2.25 ± 2.05, *p* = 0.0135) (Figure 2C). Based on these observations, we analyzed neuro‐cardiac interaction at 10–15 days in co‐cultures in optogenetic evaluations.

### 3.3 Optogenetic Modulation of Sensory Neuron‐Cardiomyocyte Interactions

To validate the specific interaction between the DRG^HS^ neurons and cardiomyocytes, we employed an optogenetic strategy in which ChR2 and GCaMP6s expression virus transduced neonatal cardiomyocytes, stimulated with 488 nm blue light stimulation to evaluate Ca^2+^ activity of GCaMP6s‐expressing DRG^HS^ neuron in coculture. The expression of ChR2 in cardiomyocytes was traced with the reporter RFP signal, and GCaMP6 biosensor expression in both populations was validated by reporter GFP expression under confocal microscopy (Figure 2D). The cardiomyocyte in cocultures was stimulated with 0.2 Hz or 1 Hz blue light, enhancing Ca^2+^ activity in DRG^HS^ neurons (Figure S2A). The 1 Hz stimulation of cardiac cells led to higher amplitude in Ca^2+^ spikes indicative of a robust response that gradually recovered to the resting state in DRG^HS^ neurons (Figure S2B). The 1 Hz optogenetic stimulation of cardiomyocytes induced rhythmic, high frequency, and high amplitude Ca^2+^ spikes compared to baseline activity (Figure 2E). The DRG^HS^ neurons exhibited synchronized, frequent and strong Ca^2+^ activity spikes during the optogenetic stimulation of the interacting cardiomyocytes (Figure 2F,G). Optogenetic stimulation of cardiomyocytes in cocultures induced Ca^2+^ activity with higher amplitudes (96.06 ± 157.3 and 60.61 ± 130.18, *p* = 0.007) in DRG^HS^ compared to baseline activity (Figure 2G, Figure 2H, left panel). Correlation analysis revealed that there was a trend showing an increase in amplitude of Ca^2+^ signals following optogenetic stimulation and that neurons with lower baseline fluorescent intensity showed higher Ca^2+^ signals (Figure 2H, right panel; *r* = 0.242, *p* = 0.22). Furthermore, spike duration of DRG^HS^ lasted longer compared to its baseline duration (13.09 ± 22.66 and 6.16 ± 10.03, *p* = 0.049) following optogenetic stimulation interacting cardiomyocytes (Figure 2I). Lastly, the number of events in the neurons at rest (1.53 ± 3.47) and after cardiomyocyte stimulation (3.38 ± 8.41) were measured to have a high correlation relative to baseline activity (*r* = 0.798, *p* < 0.0001) (Figure 2J).

Beside optogenetic stimulation, we chemically induced cardiomyocytes with the β‐Adrenergic agonist isoproterenol and evaluated DRG^HS^ activity to model/replicate the sensory transmission route. Following isoproterenol stimulation, mono and coculture cardiomyocytes showed enhanced contraction frequency as reported previously (Figure S3A) (Ginsburg and Bers 2004). The DRG^HS^ neuronal activity was comparatively evaluated for the number of events, amplitude, and the duration of the firing in the absence or presence of cardiomyocytes induced by isoproterenol. DRG^HS^ monocultures did not display spontaneous activity overall, except 2 cells in the recording of 54 neurons (Figure S3B, upper panel). Similarly, in DRG^HS^ alone cultures, the quiescent neurons did not respond to isoproterenol stimulation regarding the number of events (Figure S3C), amplitude (Figure S3D), and duration (Figure S3E) of Ca^2+^ signal. In contrast, the DRG^HS^ neurons in cocultures with cardiomyocytes showed spontaneous Ca^2+^ spikes (Figure S3B, lower panel). Following isoproterenol administration, a subset of neurons exhibited heterogeneous responses: 31.03% and 20.69% of neurons showed increases in amplitude and event frequency, respectively, while 17.24% and 24.13% exhibited decreases in these parameters (Figure S3C–E). Although the opposing directions of these responses resulted in no net change during comparison and correlation analysis, relative changes in the activity of DRG^HS^ in cocultures showed a significant increasing trend in the firing frequency, amplitude, and duration of firings compared to DRG^HS^ monoculture (Figure S3C–E).

### 3.4 Global Gene Expression Profiling of Heart‐Specific Sensory Neurons

**FIGURE 3:**
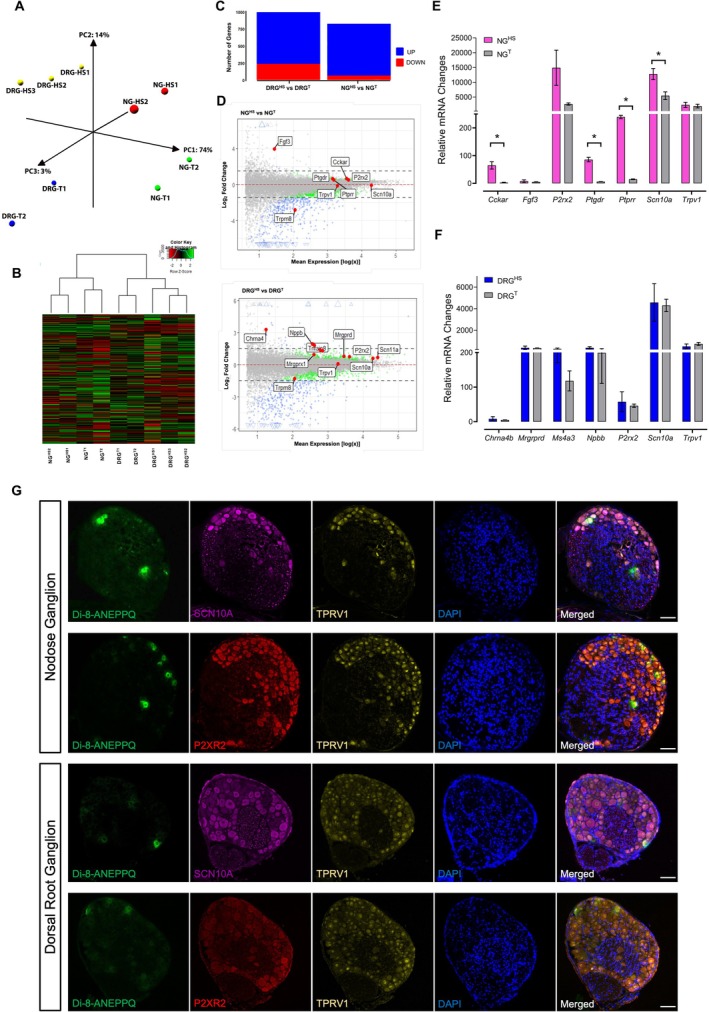
Global transcriptome analysis of DRG^HS^ and NG^HS^ populations with molecular validation. (A) PCA of total gene expression profiles illustrating the distinct separation between DRG and NG‐derived samples (PC1: 74%) and between heart‐specific versus total sensory populations (PC2: 14%). Variations within either DRG or NG cardiac populations were relatively low, as PC3: 3%. (B) Hierarchical clustering displaying the total number of differentially expressed genes. (C) The up‐regulated and down‐regulated genes selected based on the criteria of |log2FC| > 0.5, padj < 0.05 were highlighted in red and blue, respectively, in the bar graph. (D) MA plots displaying the distribution of total and selected up‐regulated and down‐regulated genes in DRG^HS^ and NG^HS^ samples. For DRG, gray dots (*p*_adj_ > 0.05); green dots (*p*_adj_ < 0.05; lfc < 1.5, *n* = 658); blue dots (*p*_adj_ < 0.05; lfc > 1.5, *n* = 413). For NG, gray dots (*p*adj > 0.05); green dots (*p*_adj_ < 0.05; lfc < 1.5, *n* = 432); blue dots (*p*adj < 0.05; lfc > 1.5, *n* = 411). Validation of selected differentially expressed genes using qRTPCR analysis, showing relative mRNA changes in (E) NG^HS^ and (F) DRG^HS^ neurons compared to total populations. All values denoted mean ± S.D.; *p < 0.05, otherwise nonsignificant. (G) Representative immunofluorescence images of Di‐8‐ANEPPQ‐labeled neurons (green) in tissue section of NG and DRG. Tissue section co‐staining for SCN10A (magenta), TRPV1 (yellow), P2XR2 (red), and the nuclei were stained with DAPI (blue). Scale bars: 100 μm. PCA: Principal component analysis, MA: The logged intensity ratio (M) versus the mean‐logged intensity (A), lfc, logarithmic fold change, log2FC, logarithmic fold change.

To determine whether DRG^HS^ and NG^HS^ have a distinctive transcriptional profile compared to the total population, the global transcriptome analysis of retrogradely labeled heart‐innervating sensory neurons was evaluated. Total RNA extracted from FACS purified DRG^HS^ and NG^HS^ from labeled tissue (Figure 1A) together with the entire sensory neuron population of DRG^T^ and NG^T^ from vehicle‐treated SHAM control were subjected to RNA sequencing, yielding around 35‐51 × 10^6^ total reads. There were no significant differences in FPKM among samples (Figure S4A). Around 15,853 transcripts were detected in the pool of cardiac sensory neurons compared to total cells based on the gene expression profiles (Figure S4B). Principal component analysis (PCA) of the top 500 differentially expressed genes (DESeq2) revealed a clear separation between DRG‐ and NG‐derived samples along the primary component, accounting for 74% variance. Importantly, the variation within the DRG^HS^ group or NG^HS^ group was confined to only about 3% at PC3, confirming high reproducibility across biological replicates (Figure 3A). Furthermore, DRG^HS^ and NG^HS^ samples exhibited distinct clustering patterns relative to their respective total tissue samples along the secondary axis, accounting for 14% of variance. Hierarchical clustering supported the PCA results, yielding four well‐defined clusters (Figure 3B).

Differential expression analysis based on the criteria of log2FC| > 0.5, *p*adj < 0.05 identified 995 genes altered in DRG^HS^ neurons relative to DRG^T^, in which 238 genes were upregulated, and 757 genes were downregulated. For the NG population, from 827 genes in NG^HS^, only 66 genes showed an upregulation, while the majority of 761 were measured to be downregulated relative to NG^T^ (Figure 3C). The logged intensity ratio (M) versus the mean‐logged intensity (A), MA plots highlighted representative examples were selected based on high significance or functional sensorial relevance (Figure 3D). The top 50 up‐ and down‐regulated genes for both DRG and NG were demonstrated in Figure S5A,B.

Next, the RNA samples from the global transcriptional profile were validated by qRT‐PCR based on *p*‐value significance and functional relevance (Figure 3E,F). Pearson correlation analysis demonstrated strong concordance between RNA‐seq and qRT‐PCR datasets (*r* = −0.69 to −0.87; Figure S6). Expression of *Cckar*, *Ptgdr*, *Ptprr*, and *Scn10a* was significantly higher in NG^HS^ compared to NG^T^ (Figure 3E). Although *P2xr2* and *Scn10a* were also elevated in DRG^HS^ relative to DRG^T^, the differences did not reach statistical significance (Figure 3F). Moreover, the functionally relevant *Trpv1* (Transient Vanilloid Receptor1), the NG^HS^‐differentially expressed *Scn10a* (TTX‐resistant Na^+^ channel), and both DRG^HS^ and NG^HS^‐differentially expressed *P2rx2* (purinergic receptor P2X2) were validated for protein expression by immunohistochemistry. Immunofluorescence imaging of the DRG (T segment) and NG tissue sections showed an overlapping Di‐8‐ANEPPQ fluorescence with either P2RX2, SCN10A, and TRPV1, confirming mRNA expression data (Figure 3G).

Mas‐related gene family members (Mrgpr) were enriched in DRG samples relative to NG samples across the dataset. Although Mrgprd did not reach statistical significance at p adj < 0.05, the mRNA expression was validated as a candidate by our qRT‐PCR analysis (Figure S5C, Table S13). Moreover, the Mrgprd‐Cre::tdTomato transgenic mice were analyzed by the tdTomato reporter expression (Olson et al. 2017). Consistent with the transcriptome data, *Mrgprd* expression was detectable only in the DRG^HS^ tissue (Figure S7A), albeit no *Mrgprd* expression in the NG tissues (Figure S7B).

### 3.5 Pathway Analysis Differentially Expressed Genes in Heart‐Specific Sensory Neurons

**FIGURE 4:**
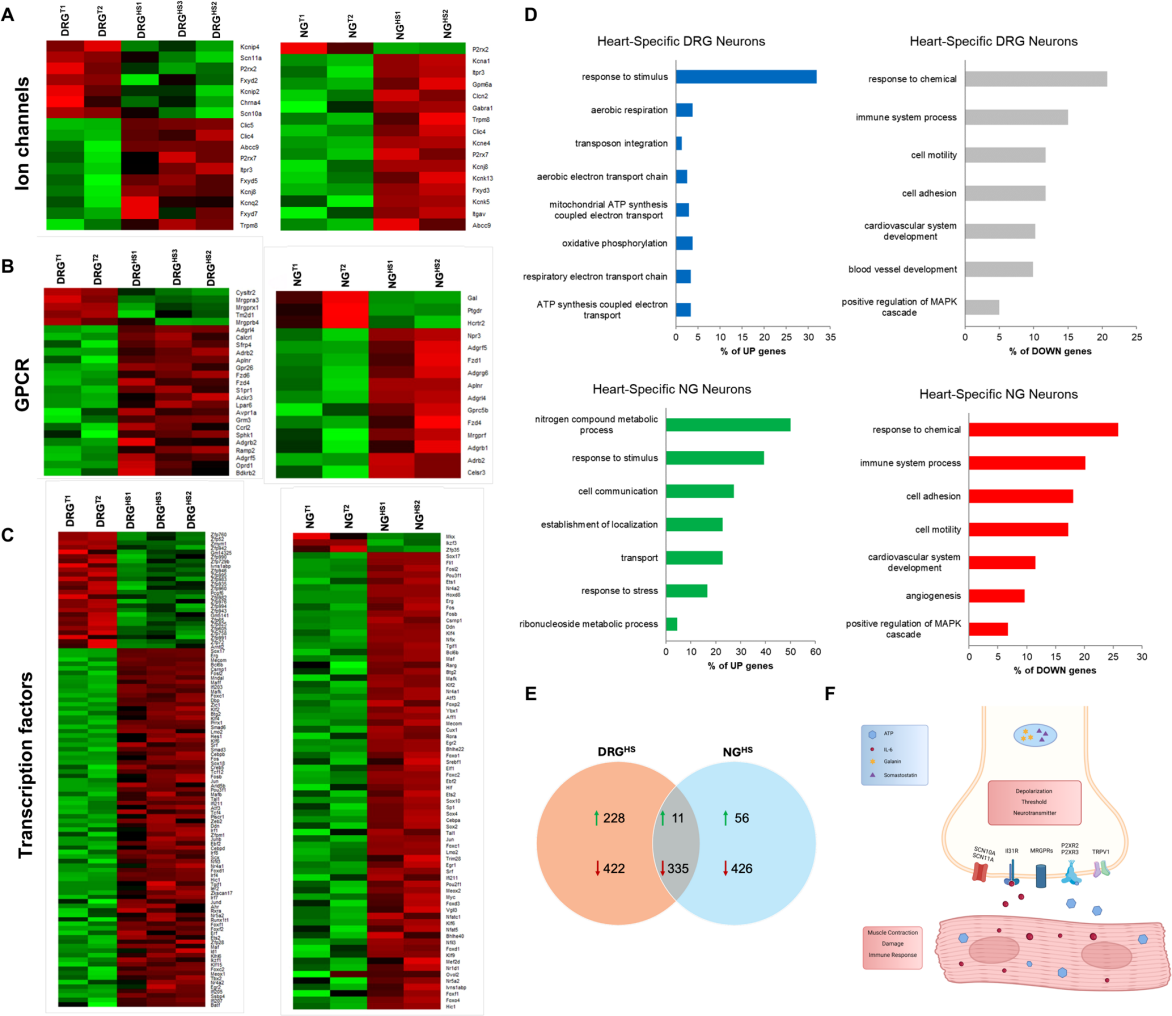
Functional classification of transcriptomic profiles from DRG^HS^ and NG^HS^ neurons. Heatmaps illustrating differentially expressed genes categorized by their functional roles as (A) ion channels, (B) GPCRs, and (C) transcription factors. Each panel compares DRG^HS^ and NG^HS^ with their respective total populations. Green and red color represent the relatively upregulated and down‐regulated genes, respectively. (D) Biological GO pathway analysis based on up‐regulated or down‐regulated genes in DRG^HS^ (upper panel) and NG^HS^ (lower panel). (E) The Venn diagram illustrated the number of mutually expressed and differently up/down regulated genes in DRG^HS^ and NG^HS^. (F) The hypothetical diagram summarizing the transcriptomic data.

To explore the functional categories of upregulated genes in DRG^HS^ and NG^HS^ neurons, we performed Gene Ontology (GO) enrichment analyses for GPCR activity (GO:0004930), ion channel activity (GO:0005216), and DNA‐binding transcription factor activity (GO:0003700) (Figure 4). Ion channel genes such as *Chrna4*, *Scn11a*, *Scn10a*, *P2rx2, Kcnip2*, *Kcnip4*, and *Fxyd2* showed relatively upregulated expression, whereas some transcripts such as *Kcnj8*, *Kcnq2*, *P2rx7*, and *Trpm8* were less abundant in DRG^HS^ neurons compared to the DRG^T^ (Figure 4A). GPCR‐related genes including *Cysltr2*, *Mrgpra3*, *Mrgprb4*, and *Mrgprx1* were enriched in DRG^HS^ (Figure 4B). In contrast, NG^HS^ neurons displayed fewer upregulated ion channel or GPCR genes, limited to *P2xr2*, *Ga*l, *Ptgdr*, and *Hcrtr2* (Figure 4A,B). A greater number of transcription factor genes were differentially expressed in DRG^HS^ neurons compared to NG^HS^ (Figure 4C), highlighting a distinct molecular identity for cardiac sensory neurons.

To reveal enriched biological processes and pathways, differentially expressed genes in cardiac innervating DRG and NG neurons were analyzed separately using the GEO and Reactome databases. GO and Reactome pathway analyses revealed that upregulated DRG^HS^ genes were enriched in a variety of pathways, including responsiveness to stimulation (GO:0050896) and mitochondrial function, ATP synthesis, and oxidative phosphorylation (GO:0042773, Figure 4D upper panel; Table S14). Downregulated DRG^HS^ genes were associated with processes including “response to chemical” (GO:0050896), “immune system process” (GO:0002376), “cell mobility/adhesion” (GO:0007155) (Figure 4D upper panel, Table S14). There were no significantly enriched GO terms found for upregulated NG^HS^ genes, though several pathways were linked to “nitrogen compound metabolic process” (GO:0006807), “response to stimulus” (GO:0050896), and “establishment of localization” (GO:0051234) (Figure 4D lower panel). Interestingly, downregulated transcripts in the NG^HS^ group showed an overlap with biological pathways to DRG^HS^ (Figure 4D; Table S15). To evaluate the similarity in gene expression profiles in DRG^HS^ and NG^HS^, the Venn diagram was constructed, identifying 11 genes commonly up‐regulated (*Far2*, *Plcl2*, *Rab39*, *P4ha2*, *P2xr2*, *Mettl16*, *Cystm1*, *Cacybp*, *Mrpl12*, *Stip1*, and *Ahsa2*) and 335 down‐regulated transcripts in both cardiac DRG and NG sensory neurons (Figure 4E).

To classify heart‐specific sensory neurons, we selected transcripts from our top expression and aligned them with previously reported nomenclature (Zeisel et al. 2018; Kupari et al. 2019) (Figures S8 and S9). DRG^HS^ neurons primarily mapped to non‐peptidergic clusters, particularly PSNP6 (Figure S8), characterized by *P2rx2*, *Sst*, *Nppb*, and *Il3ra* expression (Usoskin et al. 2015). In addition, almost all transcripts except for *Trpv1* and *Scn10a* were assigned to a distinct group, matching with nociceptor‐like molecular signature (Figure S8). NG^HS^‐enriched transcripts such as *Lypd6* and *Hctr2* corresponded to the NG3 cluster defined by Kupari et al. (2019) (Figure S9). On the other hand, NG^HS^ neurons share similarities with all subgroups (Kupari et al. 2019) except *Lypd6* and *Hctr2* genes which are elusive to the NG3 cluster (Figure S9). Therefore, the *Hctr2* and *Lypd6* genes can be utilized to access and functionally characterize this cluster.

## 4 Discussion

We provided data to distinguish heart‐innervating sensory neurons of spinal or vagal origin. Here, retrogradely labeled and FACS‐purified DRG or NG neurons innervating the heart formed functional interactions with neonatal cardiomyocytes in coculture. Electron microscopy analysis demonstrated that DRG^HS^ or NG^HS^ neurons were physically interacting with cardiomyocytes by branching and forming extensions, forming multiple point contacts with the underlying cardiomyocyte sarcolemma. NG^HS^ processes demonstrated robust anchoring onto the cardiac membrane. These ultrastructural observations confirmed the presence of sub‐micron membrane appositions. These observations are consistent with previous reports demonstrating skeletal muscle cells interacting with sympathetic, parasympathetic, or sensory neurons in cocultures (Takeuchi et al. 2011; Hick et al. 2013; Oh et al. 2016). Interestingly, the spontaneous activity or optogenetic stimulation of the DRG^HS^ increased significantly when cocultured with cardiomyocytes, compared to monocultures. Neuronal activity in cocultures sustained over time, although Ca^2+^ dynamics in cardiomyocytes showed some alterations, potentially due to fibroblast overgrowth consistent with the literature (Nguyen et al. 2012; Wells et al. 2019; Alegret et al. 2023). Furthermore, following optogenetic activation of ChR2‐expressing cardiomyocytes, neuronal Ca^2+^ activity in coculture was correlatively enhanced, which returned to baseline whenever the blue light was switched off, confirming functional coupling. These observations are consistent with the previously reported cardiomyocyte‐sympathetic neuron and neuron‐endocrine cells interaction studies (Kaelberer et al. 2018). Notably, stimulation of β‐adrenergic signaling induced heterogeneous neuronal responses in amplitude, duration, and number of firings. This observation may stem from the fact that isoproterenol altered neuronal excitability threshold (Pluteanu et al. 2002) and increased activity in cardiomyocytes desensitizing DRG neurons (Koplas et al. 1997; Matsushita et al. 2018). There are extremely limited studies regarding sensory neuron‐cardiomyocyte interactions, implying mechanotransduction, paracrine, and/or metabolic signaling mechanisms, based on the literature. In one study, the vagal sensory neurons were shown to respond to heart volume changes through mechanosensation (Liu et al. 2026). In other studies, Angiotensin II, Atrial Natriuretic Peptide (Sadoshima et al. 1993; Church et al. 2000; Moreira et al. 2009; Li, Wu, et al. 2016), and metabolites such as protons and lactate (Kaijser and Berglund 1992; Light et al. 2008; Lyu et al. 2022) secreted from the cardiac tissue were implicated in regulating neural response, since sensory neurons express specific receptors.

Transcriptomic profiling revealed a clear separation between heart‐innervating and total sensory neuron populations with respect to ion channels and GPCRs, highlighting anatomical specialization. Notably, NG^HS^ neurons showed a higher number of downregulated transcripts than DRG^HS^ neurons, suggesting that transcriptional suppression may contribute to the specialization of the cardiac vagal route. Among the significant transcripts were *P2xr2*, *Ahcy*, and *Adk*, consistent with the established release of ATP and adenosine from cardiac tissue under steady state or in response to ischemic damage (Fu and Longhurst 2010; Burnstock 2017b). TRPV1, a non‐selective cation channel, is sensitive to a variety of stimuli, including heat, pH, and capsaicin (Caterina et al. 1997; Dhaka et al. 2009). In sensory neurons, the TRPV1 channel is responsible for pain sensation, hence its expression is predominantly restricted to nociceptive neurons (Zeisel et al. 2018; Kupari et al. 2019). Although TRPV1 expression is not exclusive to heart‐specific sensory neurons, its known role in cardiac sympathetic tone (Zahner et al. 2003; H.‐J. Wang et al. 2014, 2017) and its expression in DRG^HS^ and NG^HS^ neurons (Akgul Caglar, Durdu, et al. 2021) support its functional relevance. Consistent with a recent study showing that co‐activation of TRPV1 and P2XR2 vagal sensory neurons innervating the lower airways induces bradycardia and bradypnea (Patil et al. 2025), co‐expression of TRPV1 and P2XR2 channels in DRG^HS^ and NG^HS^ neurons could suggest a role in cardiac nociception (Burnstock 2017a; Gao et al. 2007; Wang et al. 2009) (Figure 4F). In support of this, the average DRG^HS^ soma diameter was found to be around 20 μM, consistent with small‐diameter sensory neurons (Price 1985; Li, Li, et al. 2016) often classified as nociceptive. A recent study further demonstrated that TRPV1‐innervating VSN neurons increased following myocardial infarction (MI), and that ablation of TRPV1 ameliorates MI‐associated pathologies, including improved cardiac conduction, reduced infarct expansion, and enhanced angiogenesis (Yadav et al. 2026). Moreover, VSN neurons form a functional loop with the paraventricular nucleus and the superior cervical ganglion, thereby contributing to cardiac regulation (Yadav et al. 2026).

DRG^HS^ neurons displayed enriched mRNA transcripts for Mas‐related GPCRs (*Mrgpr*), including *Mrgprd*, *Mrgrpa3*, and *Mrgrpx1*, compared to DRG^T^. Further confirmation in the Mrgprd transgenic model, our data confirmed expression specifically in DRG tissue. MRGPR‐related receptors have defined roles in nociception, itch sensation (Dong et al. 2001; Liu et al. 2009, 2012; Han et al. 2013; Warwick et al. 2021), and neuron‐immune interactions (Shi et al. 2015; Niccoli et al. 2018). Based on the reported literature and our findings, we speculate that MRGPR+ spinal sensory neurons may participate not only in pain transmission but also in immune regulation in the cardiac system (Figure 4F). In support of this, immune regulatory transcripts, such as *Il31ra*, *Tnfa8*, *Ptges3*, *Nppb*, and *Somatostatin*, were also enriched in DRG^HS^ neurons, emphasizing the immuno‐neuro regulation in heart function. In response, immune mediators were reported to sensitize sensory neurons through TRPV1 and SCN10A, thereby reducing threshold during inflammation or injury (Chavan et al. 2017; Pinho‐Ribeiro et al. 2017) (Figure 4F). Similarly, heart‐specific neurons may modulate neutrophil and T‐cell responses in the heart (Baral et al. 2018; Almanzar et al. 2025). In another study, the stellate (cervical sympathetic) ganglion is not only responsible for transmitting motor output to the heart but also plays a role in regulating the immune response following MI. Accordingly, inhibition of IL‐1β in the stellate ganglion ameliorated MI‐associated pathology and improved cardiac function, underscoring the importance of immune–neural regulation (Yadav et al. 2026).

Several receptors, such as orexin receptor 2 (*Hcrtr2*) in DRG (Jiao et al. 2024) and neuropeptide Y receptor Y2 in NG (Lovelace et al. 2023), were previously implicated in cardiac reflex regulation, which may potentially contribute to heart‐brain communication. Interestingly, our data revealed that orexin receptor 2 (*Hcrtr2*) is highly expressed in the NG^HS^ (Figure 4B), in parallel to its expression shown by immunohistochemistry in DRG tissue previously (Jiao et al. 2024). Our transcriptomic analysis further revealed NG^HS^ neurons exhibit elevated expression of Prostaglandin D2 Receptor (*Ptgdr*), cholecystokinin A receptor (*Cckar*), and protein tyrosine phosphatase receptor type R (*Ptprr*), consistent with recent single‐cell RNA analysis (Zhao et al. 2022), thereby supporting the consistency with our bulk RNA‐seq approach. Prostaglandins released at myocardial ischemia‐induced cardiac tissue damage are known to activate vagal afferents and suppress sympathetic outflow (Zucker et al. 1989; Ustinova and Schultz 1994). Thus, the upregulated expression of *Ptgdr* in NG^HS^ neurons may implicate a role in autonomic balance in cardiac tissue. Another NG^HS^ prominently expressed receptor *Cckar* was shown to respond to cholecystokinin, a neuropeptide involved in satiety, nociception, and heart rate regulation (Kaczyńska and Szereda‐Przestaszewska 2015; Koizumi et al. 2020; Arnold et al. 2024). Notably, cholecystokinin signaling via TRPV1 channels in NG neurons was reported (Arnold et al. 2024), indicating a potential mechanism by which metabolic or inflammatory signals could modulate cardiac sensory input. Supporting this notion, Arnold et al. demonstrated that activation of CCKAR in the NG was shown to slow respiratory rhythm and to reduce tidal volume (Arnold et al. 2024), further underscoring its role in autonomic regulation.

Studies in cerebellar purkinje cells showed PTPRR regulates mitogen‐activated protein kinase activity and neuronal signaling pathways. Although there is no direct evidence currently linking *Ptprr* to NG function, the receptor upregulation in NG^HS^ neurons may imply a neuro‐cardiac interaction. High levels of growth factor 3 (*Fgf3*) have been implicated in cardiac development (Urness et al. 2011) and in hypersensitization of DRG through the Akt/mTOR pathway following neuronal injury (Guo et al. 2024). These findings suggest that significant upregulation of *Fgf3* in NG^HS^ may exert neuromodulator or sensitizing effects in cardiac innervating vagal neurons.

Nodose‐jugular complexes expressing the mechanoreceptor *Piezo2*, forming specialized claw‐like endings around the aortic arch were shown to be involved in baroreflex control (Zeng et al. 2018). Moreover, the ventricular wall was shown to have only a small subset of PIEZO2‐expressing cardiac mechanoreceptors, compared to the vessel components (Liu et al. 2026). In line with these findings, our data revealed a downregulation of *Piezo2* in purified NG^HS^ neurons, implying involvement of receptor activity even though its expression is relatively low across the neuronal subpopulations.

Throughout the study, female BALB/c mice were used to characterize the HS‐sensory neurons. While the molecular identity of sensory clusters has been generally consistent across sexes in the broader literature (Kupari et al. 2019), future studies utilizing male cohorts would be essential to investigate potential sex‐specific differences in sensory neuron populations innervating the heart.

## 5 Conclusion

Our findings demonstrated that sensory neurons specifically innervating the heart formed functional connections with neonatal cardiomyocytes in vitro, as shown by immunolabeling, electron microscopy, and optogenetic manipulation. Notably, DRG^HS^ cocultured with cardiomyocytes showed increased spontaneous Ca^2+^ activity and functional coupling validated by optogenetic stimulation. Moreover, cardiac sensory neurons in DRG and NG exhibited unique gene expression profiles in ion channels, GCPR, and transcription factors. Interestingly, aligning our data with published single‐cell NG and DRG RNA‐seq analysis indicated that cardiac‐specific sensory neurons have peptidergic or non‐peptidergic nociceptor‐like nature in a large subset. Collectively, our data provided a molecular framework for future investigations into heart function through sensory neurons, an area that is largely underestimated.

## 6 Limitations

The cardiac sensory neurons were identified through retrograde labeling following tracer injection into the apex of the heart. Although this approach enabled selective enrichment of heart‐projecting neurons, the restricted injection site may have limited comprehensive profiling of sensory afferents innervating other cardiac regions. Consequently, neuronal populations projecting to atrial or other ventricular areas may be underrepresented.

Despite the robust molecular signatures identified, we acknowledge the statistical constraints inherent in the sample size of the study. Due to the surgical complexity and low survival rate associated with retrograde labeling, samples were pooled into biological replicates to maximize RNA input and minimize individual variability. While this strategy improves the reliability of transcript abundance estimates, it reduces the degrees of freedom for statistical testing, which may contribute to occasional discrepancies between qRT‐PCR results and RNA‐seq trends. In future investigations, employing single‐cell RNA‐seq on heart‐specific spinal and vagal sensory neurons could be utilized to circumvent the need for pooling.

Although retrograde labeling and sorting initially yielded a highly enriched neuronal population with negligible non‐neuronal mRNA contamination, non‐neuronal nuclei were observed at later culture time points. The possible reason for this observation is co‐isolation of satellite glial cells, which tightly envelop DRG and NG neuronal somata and may have been sorted together with the labeled neurons. Under in vitro conditions, satellite cells may subsequently detach, migrate, and proliferate, contributing glia contamination. However, transcriptomic analyses showed that lower expression of glial fibrillary acidic protein (*Gfap*) and glutamine synthetase (*Glul*) gene data in heart‐specific DRG and NG neurons compared to the total ganglion samples, suggesting that glial contamination from in freshly isolated heart‐specific neurons was minimal. In future studies, spinal and vagal specific markers such as Vglut2 (spinal) and Phox2b (nodose) transgenic animal approaches would provide more precise evaluation for cardiac sensory neuron populations.

Due to the limited number of Di‐8‐ANEPPQ labeled NG^HS^ neurons obtained after sorting, functional Ca^2+^ imaging in the presence and absence of optogenetic/chemical stimulation were primarily relied on DRG^HS^ neurons to investigate Ca^2+^ coupling between cardiomyocyte and heart‐specific sensory neurons. This constraint limited our ability to directly assess and compare the functional connection of heart‐specific NG neurons with cardiomyocytes.

Finally, although this study identified distinct membrane channel proteins enriched in cardiac sensory neurons, we did not perform real‐time functional imaging experiments to directly stimulate or interrogate the activity of these channels in mono and coculture systems. Therefore, the physiological relevance and functional contribution of these candidate genes remain to be established in future studies.

## Author Contributions

T. Akgul Caglar: conceptualization, data curation, methodology, visualization, and writing – original draft. Y.E. Kazci: data curation, methodology, visualization, and writing – original draft. Z.B. Durdu: data curation, methodology, and visualization. E. Vatandaşlar: Data curation, methodology, and visualization. S. Sahoglu Goktas: data curation, methodology, visualization, and writing – original draft. S. Bay: data curation, and methodology. M.U. Turhan: visualization. G. Ozturk: conceptualization and methodology. E. Cagavi: conceptualization, methodology, supervision, funding acquisition, project administration, writing – original draft, and writing – review and editing.

## Funding

This study was supported by the Scientific and Technological Research Council of Turkey (TUBITAK) under the 1001 Scientific and Technological Research Projects Funding program with grant number 115S381 and Istanbul Medipol University Scientific Research Projects Commission grant number BAP 2018/19.

## Ethics Statement

All experiments on animals were approved by the Animal Research Ethics Committee of Istanbul Medipol University and conducted in the animal research facility of Medipol Research Center (MEDITAM) under approval number 38328770‐30.

## Conflicts of Interest

The authors declare no conflicts of interest.

## Supporting information


**Figure S1:** Gating strategy and immunocytochemical validation of sorted sensory neurons. (A) Cell viability analysis of DRG (top) and NG (bottom) via flow cytometry. Necrotic cell and nuclei were stained with PI and Hoechst 33342, respectively, following enzymatic cell dissociation and/or percoll gradient. (B) Representative flow cytometry plots showing the gating strategy for DRG (top) and NG (bottom) samples. The axis displayed forward scatter (FSC) versus side scatter (SSC), pulse width for doublet exclusion, and finally the “Single Cells” selection. (C) Immunofluorescence images of FACS‐sorted Di‐8‐ANEEPQ fluorescent cells immunolabeled with TUJ‐1 (red) and DAPI (blue) at the matching field. Scale bars: 50 μm.

**Figure S2:** Optogenetic modulation of CMs and correlated neuronal Ca^2+^ activity in DRG^HS^ cocultures at resting and recovery states. (A) The coculture of DRG^HS^ expressing GCaMP6s (green) and cardiomyocytes expressing ChR2 (red) and/or GCaMP6s (green) under the confocal microscopy. Blue light stimulation of CM at increasing frequencies (Resting, 0.2 Hz, and 1 Hz) demonstrated a frequency‐dependent increase in calcium transients in DRG^HS^ neurons. (B) The representative fluorescence image of DRG^HS^ neurons expressing GCaMP6s (green) cocultured with CM expressing GCaMP6s (green) and/or ChR2 (red). The Ca^2+^ traces showed baseline activity (Resting), response to 1 Hz stimulation of CM, and the return to spontaneous activity patterns during the Recovery phase on DRG^HS^ sensory neurons. Scale bar: 50 μm.

**Figure S3:** Isoproterenol stimulation of Ca^2+^ transients in CM‐DRG^HS^ cocultures. Representative of Ca^2+^ transient traces of CM (A) and DRG^HS^ neurons (B) at monocultures or cocultures at baseline and following Isoproterenol (ISO) administration. CM showed enhanced rhythmic activity in the presence of ISO. Quantitative analysis of ISO‐induced changes in the number of events (C), the amplitude (D), and the duration (E) of Ca^2+^ signals. Violin plots and correlation graphs illustrated the relative changes and baseline dependencies of neuronal responses to beta‐adrenergic stimulation in the coculture (*N* = 2, n:29). ns: non‐significant, *p < 0.05, **p < 0.01 by paired *t*‐test.

**Figure S4:** The FPKM scatter plot of heart‐specific sensory neurons. (A) The even FPKM distribution for each group. (B) The FPKM scatter plot of DRG^HS^ against DRG^T^ (upper panel) and NG^HS^ against NG^T^ (lower panel).

**Figure S5:** Top 50 up and down genes in heart‐specific and total (A) DRG and (B) NG neurons. (C) The heatmap highlighting the specific genes of interest from Table.

**Figure S6:** Regression analysis of transcriptome data and qRTPCR analysis for DRG and NG samples.

**Figure S7:** The tdTomato reporter protein imaging reporting Mrgprd expression in the DRG and NG bilateral tissue sections of Mrgprd_cre::tdTomato mice. (A) tdTomato expression before and after tamoxifen application in DRG and (B) NG sections. Scale bars: 50 μm.

**Figure S8:** Selected upregulated genes in our DRG^HS^ data exclusively overlapping with a specific spinal sensory neuron cluster out of 17 DRG neuron populations in the single‐cell dataset previously reported in Zeisel et al. (2018).

**Figure S9:** Selected upregulated genes in our NG^HS^ data exclusively overlapping with a specific vagal sensory neuron cluster out of 18 NG neuron populations in the single‐cell dataset previously reported in Kupari et al. (2019).

**Table S1:** The list for RQN values assigned to each sample sequenced.

**Table S2:** Primer list used for qRTPCR analysis.

**Table S3:** Values for cell size, neurite diameter, and neurite number of DRG and NG populations in culture.

**Table S4:** The FPKM levels of highly expressed genes as discussed in the manuscript.

**Table S5:** List of DRG^HS^ enriched GO terms and Pathways.

**Table S6:** List of NG^HS^ enriched GO terms and Pathways.

## Data Availability

The data that support the findings of this study are available on request from the corresponding author. The data are not publicly available due to privacy or ethical restrictions.
