# Supplementary figures and images for: Heart‐Specific Spinal and Vagal Afferents: Transcriptomic Signatures and Optogenetically Modulated Functional Coupling With Cardiomyocytes
Source: Compr Physiol. 2026 Jun 30;16(4):e70203. doi: 10.1002/cph4.70203 (PMC13320145; doi:10.1002/cph4.70203)

Supplementary Figure 1\_R

A

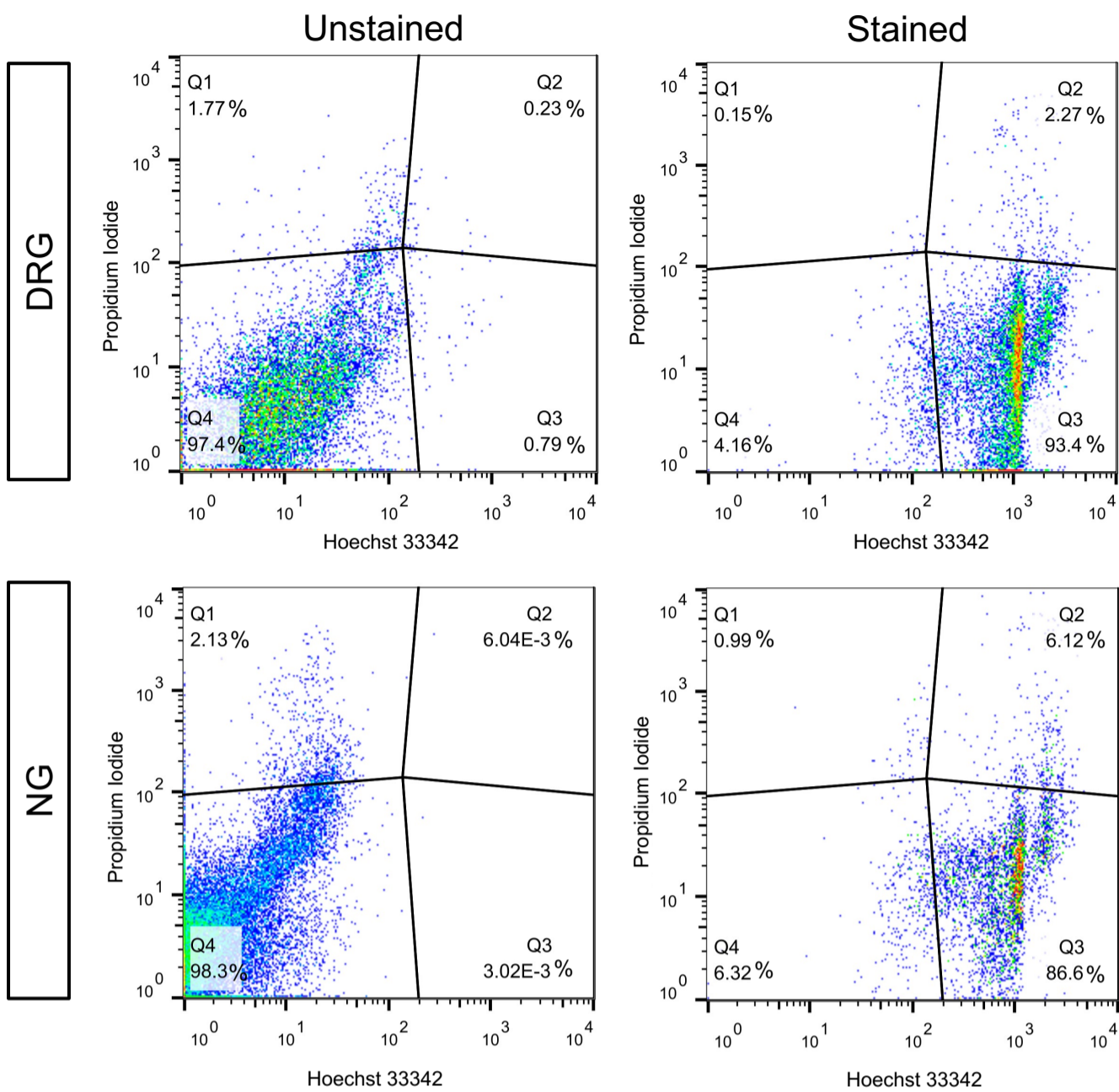

B

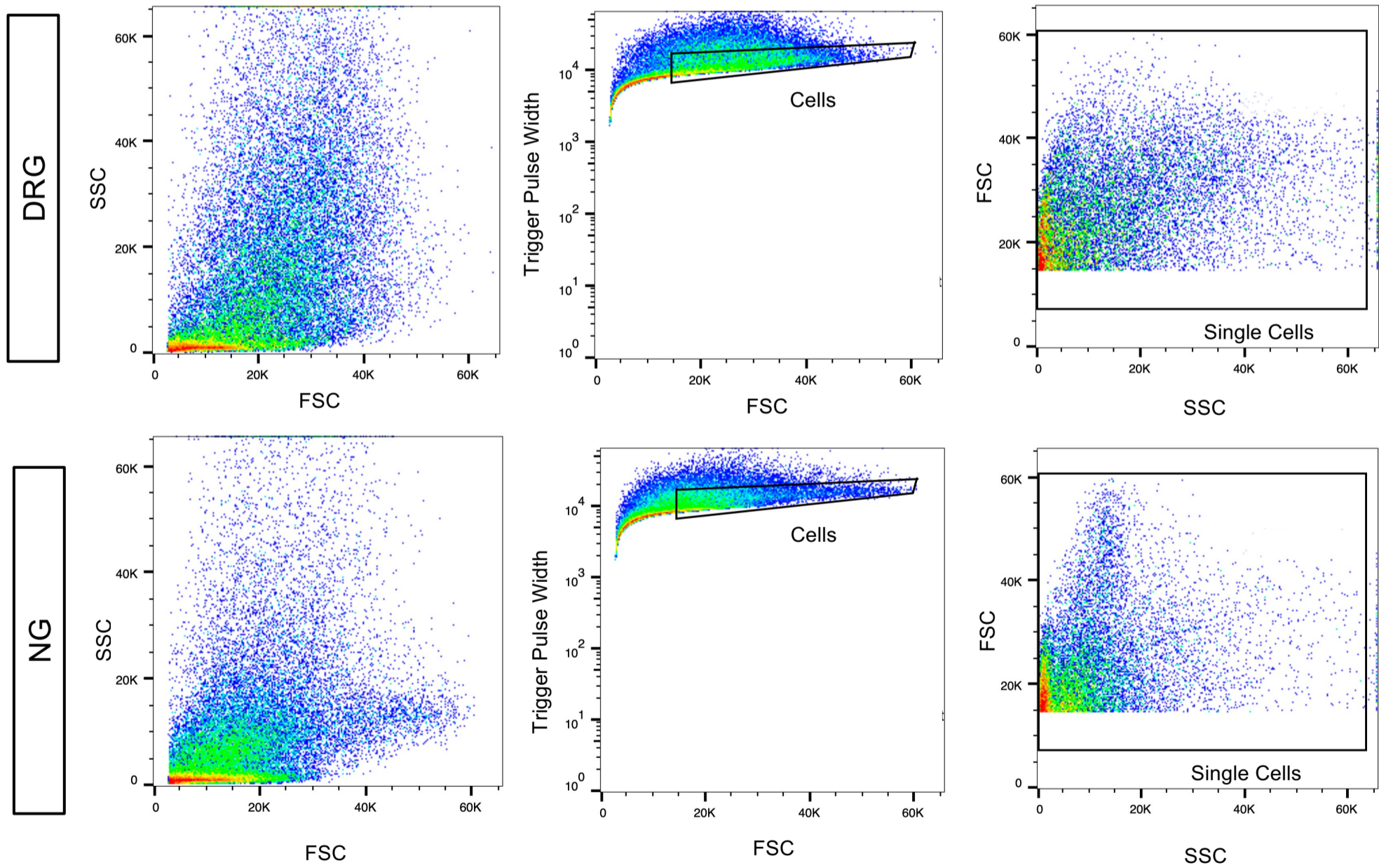

C

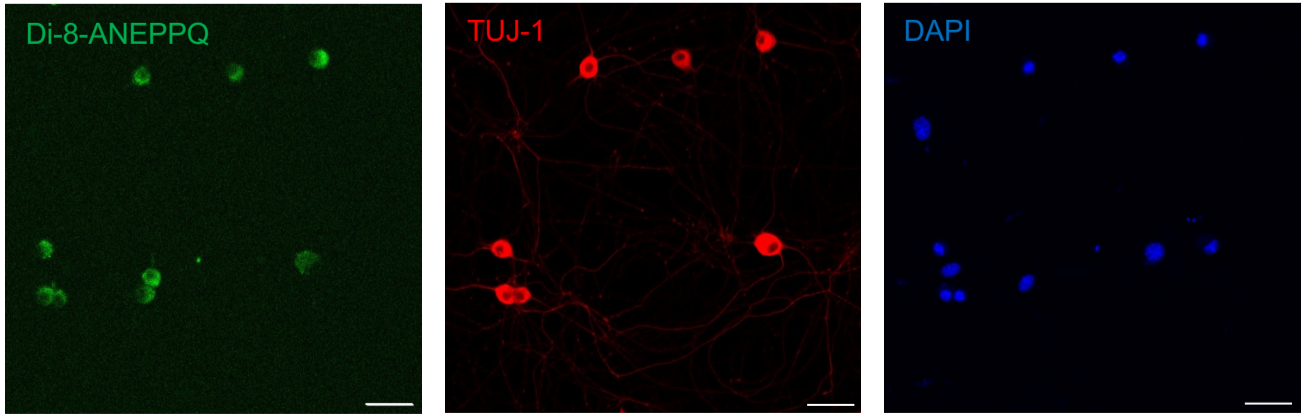

Supplement: Supplementary file 1 — Figure S1: Gating strategy and immunocytochemical validation of sorted sensory neurons. (A) Cell viability analysis of DRG (top) and NG (bottom) via flow cytometry. Necrotic cell and nuclei were stained with PI and Hoechst 33342, respectively, following enzymatic cell dissociation and/or percoll gradient. (B) Representative flow cytometry plots showing the gating strategy for DRG (top) and NG (bottom) samples. The axis displayed forward scatter (FSC) versus side scatter (SSC), pulse width for doublet exclusion, and finally the “Single Cells” selection. (C) Immunofluorescence images of FACS‐sorted Di‐8‐ANEEPQ fluorescent cells immunolabeled with TUJ‐1 (red) and DAPI (blue) at the matching field. Scale bars: 50 μm. [file CPH4-16-e70203-s011.pdf]

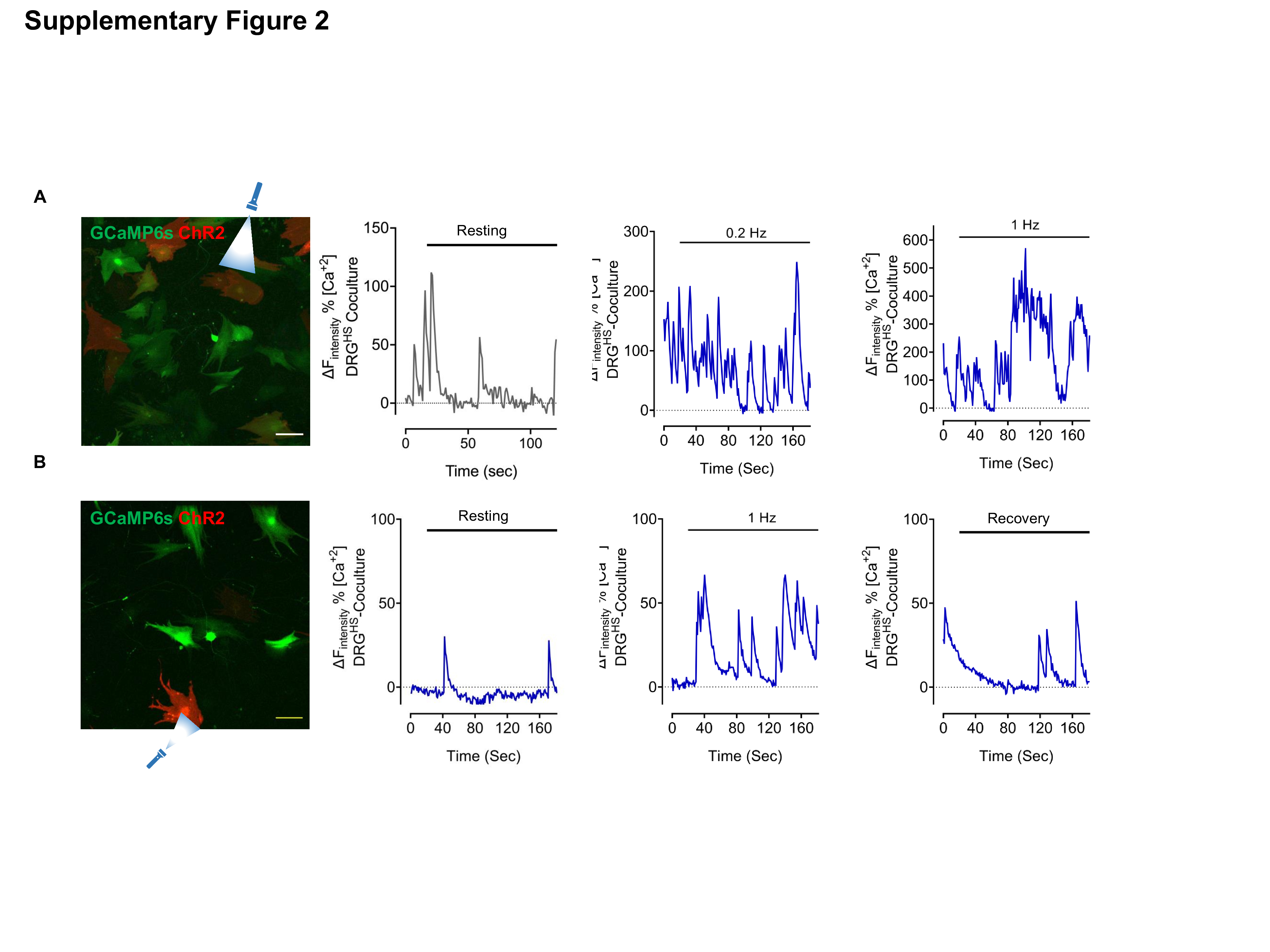

Supplement: Supplementary file 2 — Figure S2: Optogenetic modulation of CMs and correlated neuronal Ca2+ activity in DRGHS cocultures at resting and recovery states. (A) The coculture of DRGHS expressing GCaMP6s (green) and cardiomyocytes expressing ChR2 (red) and/or GCaMP6s (green) under the confocal microscopy. Blue light stimulation of CM at increasing frequencies (Resting, 0.2 Hz, and 1 Hz) demonstrated a frequency‐dependent increase in calcium transients in DRGHS neurons. (B) The representative fluorescence image of DRGHS neurons expressing GCaMP6s (green) cocultured with CM expressing GCaMP6s (green) and/or ChR2 (red). The Ca2+ traces showed baseline activity (Resting), response to 1 Hz stimulation of CM, and the return to spontaneous activity patterns during the Recovery phase on DRGHS sensory neurons. Scale bar: 50 μm. [file CPH4-16-e70203-s001.jpg]

Supplementary Figure 3\_R

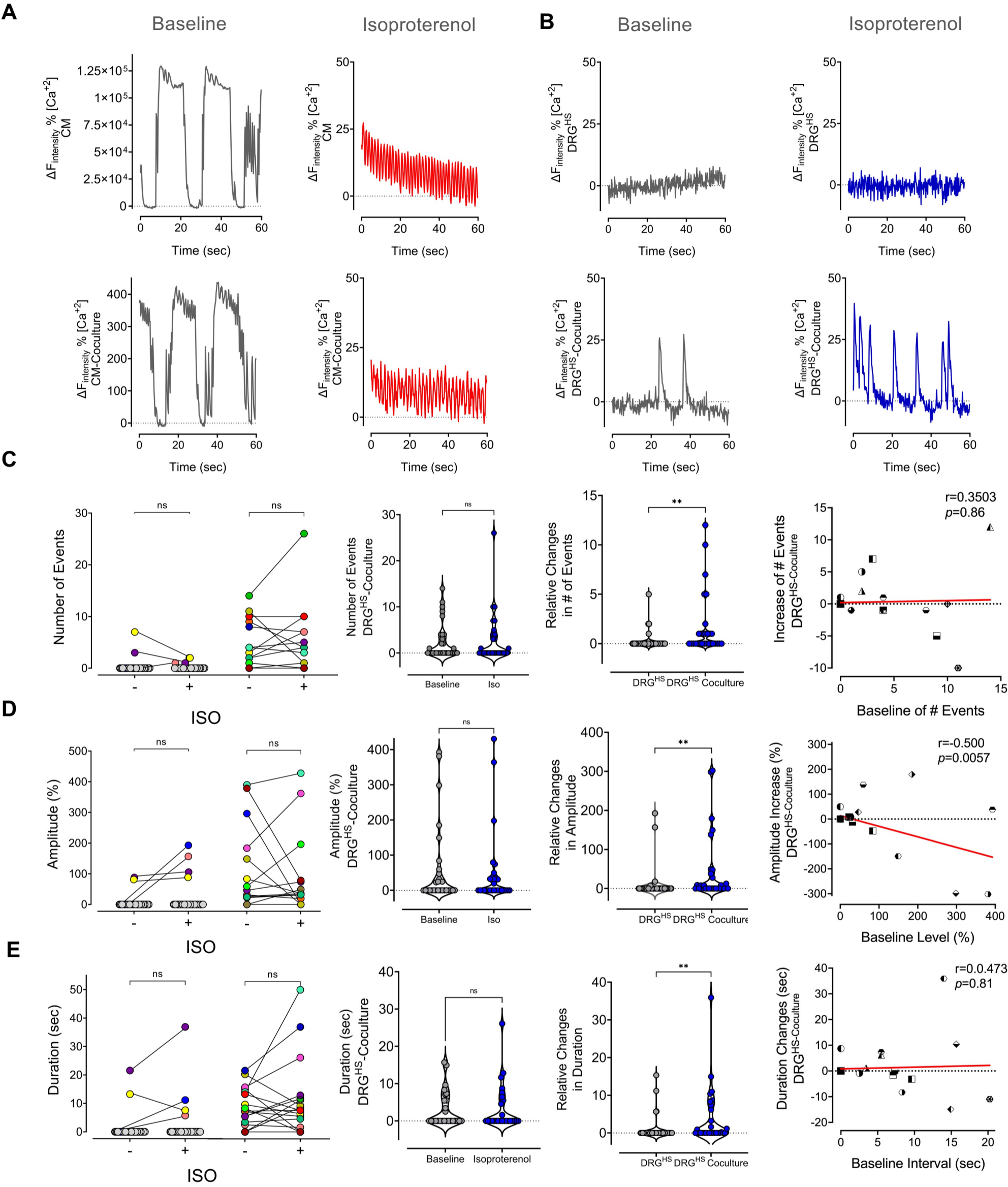

Supplement: Supplementary file 3 — Figure S3: Isoproterenol stimulation of Ca2+ transients in CM‐DRGHS cocultures. Representative of Ca2+ transient traces of CM (A) and DRGHS neurons (B) at monocultures or cocultures at baseline and following Isoproterenol (ISO) administration. CM showed enhanced rhythmic activity in the presence of ISO. Quantitative analysis of ISO‐induced changes in the number of events (C), the amplitude (D), and the duration (E) of Ca2+ signals. Violin plots and correlation graphs illustrated the relative changes and baseline dependencies of neuronal responses to beta‐adrenergic stimulation in the coculture (N = 2, n:29). ns: non‐significant, *p < 0.05, **p < 0.01 by paired t‐test. [file CPH4-16-e70203-s005.pdf]

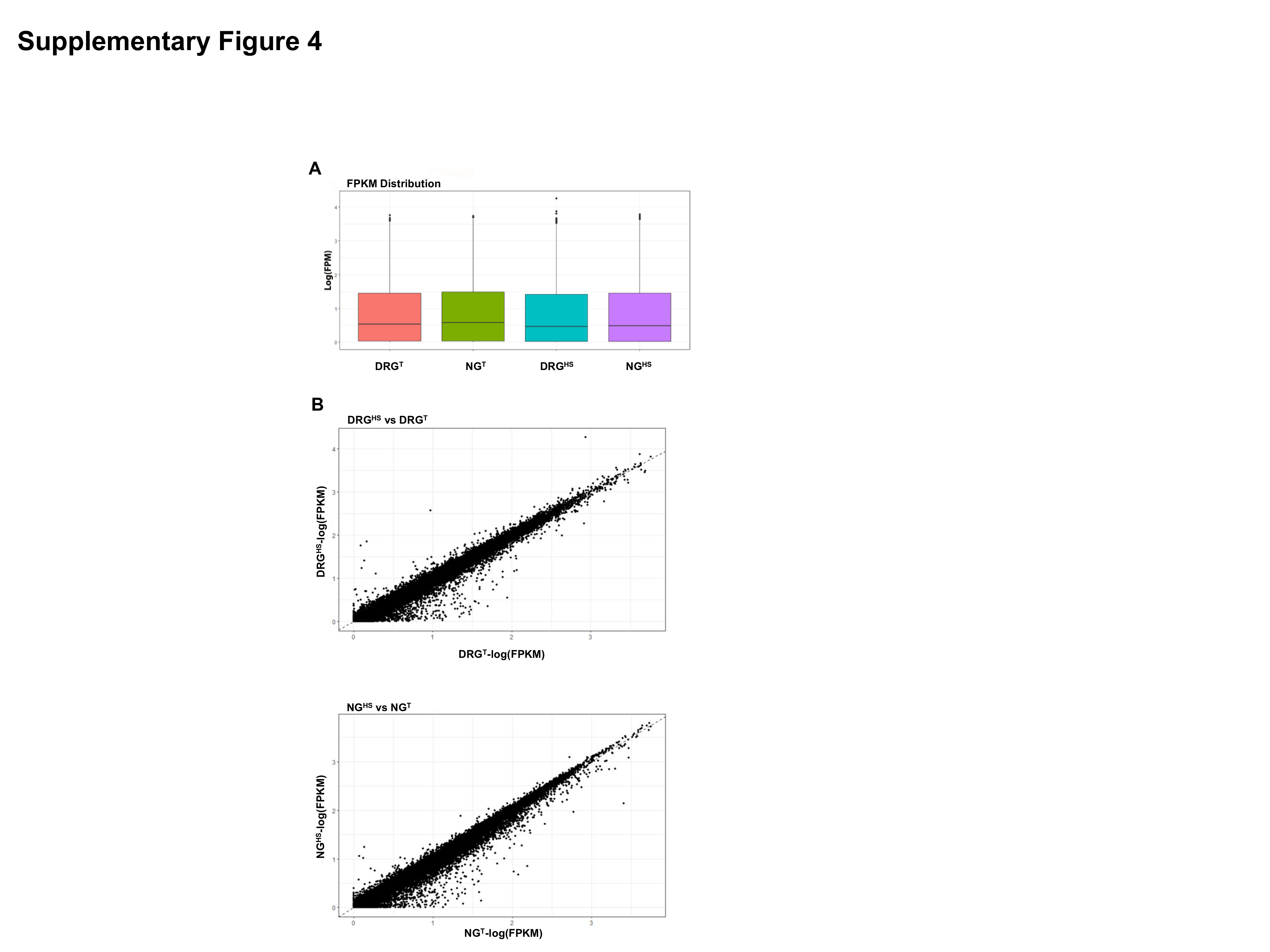

Supplement: Supplementary file 4 — Figure S4: The FPKM scatter plot of heart‐specific sensory neurons. (A) The even FPKM distribution for each group. (B) The FPKM scatter plot of DRGHS against DRGT (upper panel) and NGHS against NGT (lower panel). [file CPH4-16-e70203-s012.jpg]

Supplementary Figure 5\_R

A

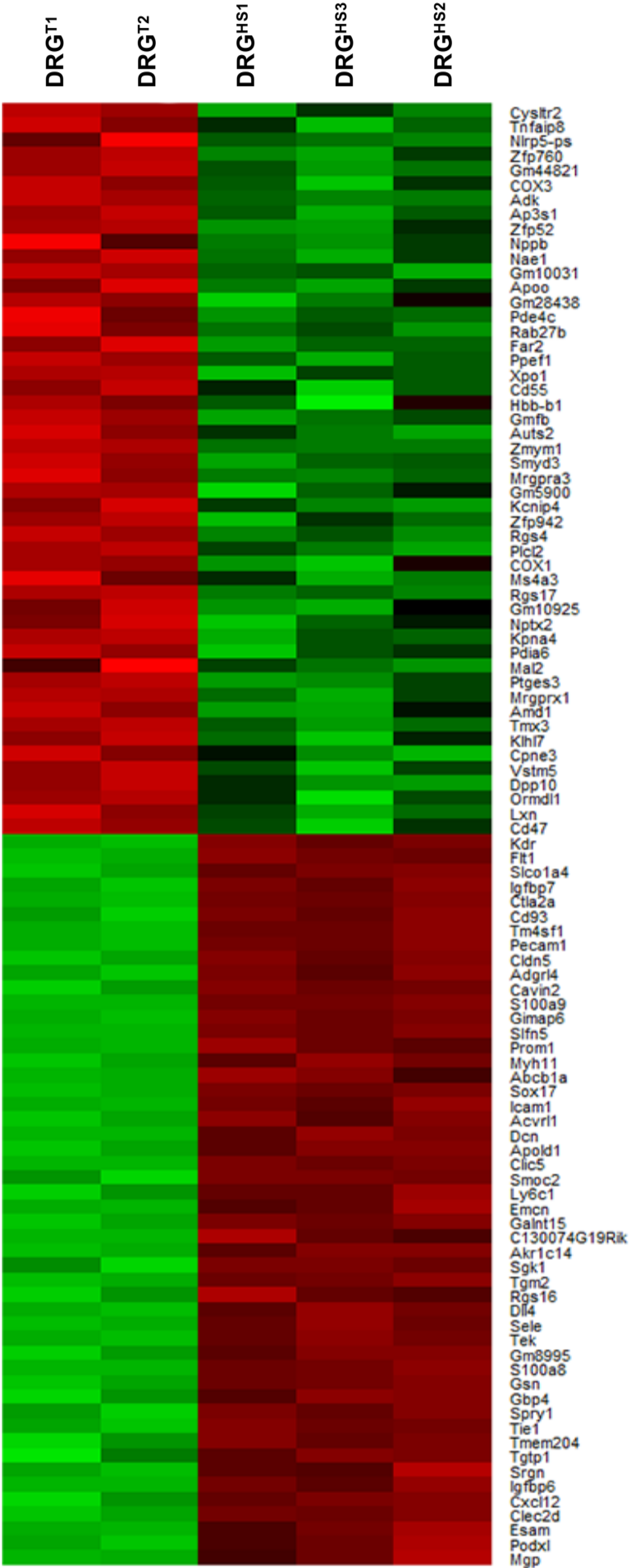

B

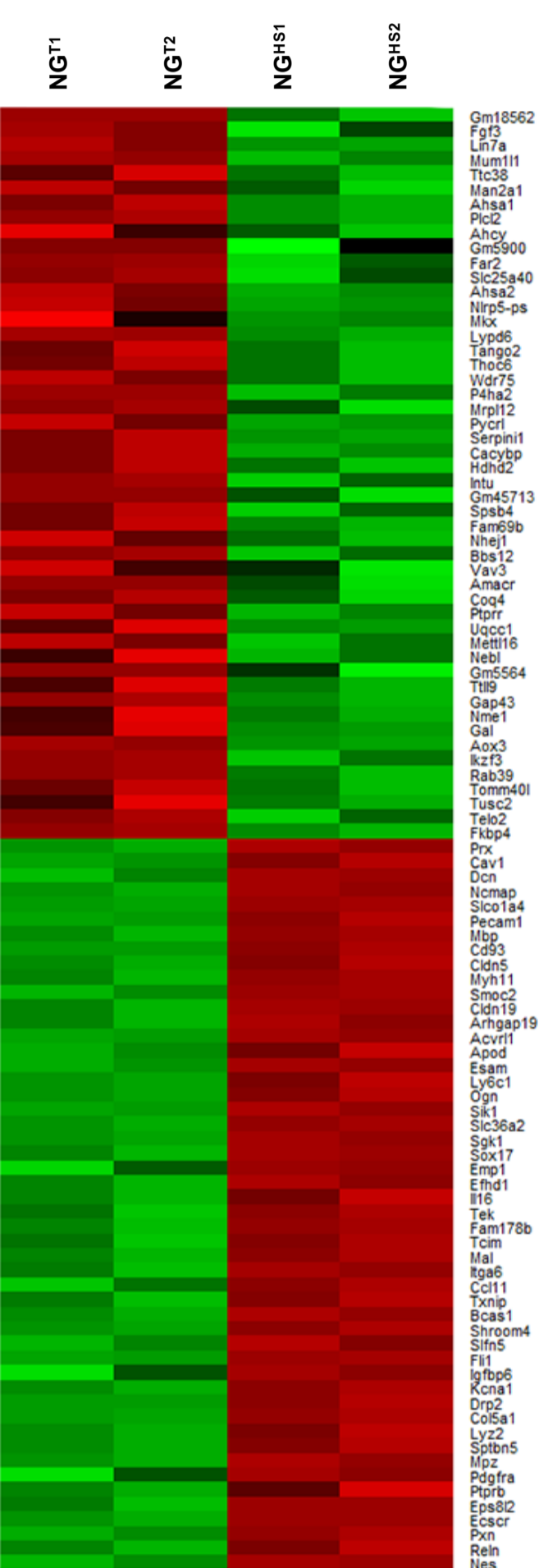

C

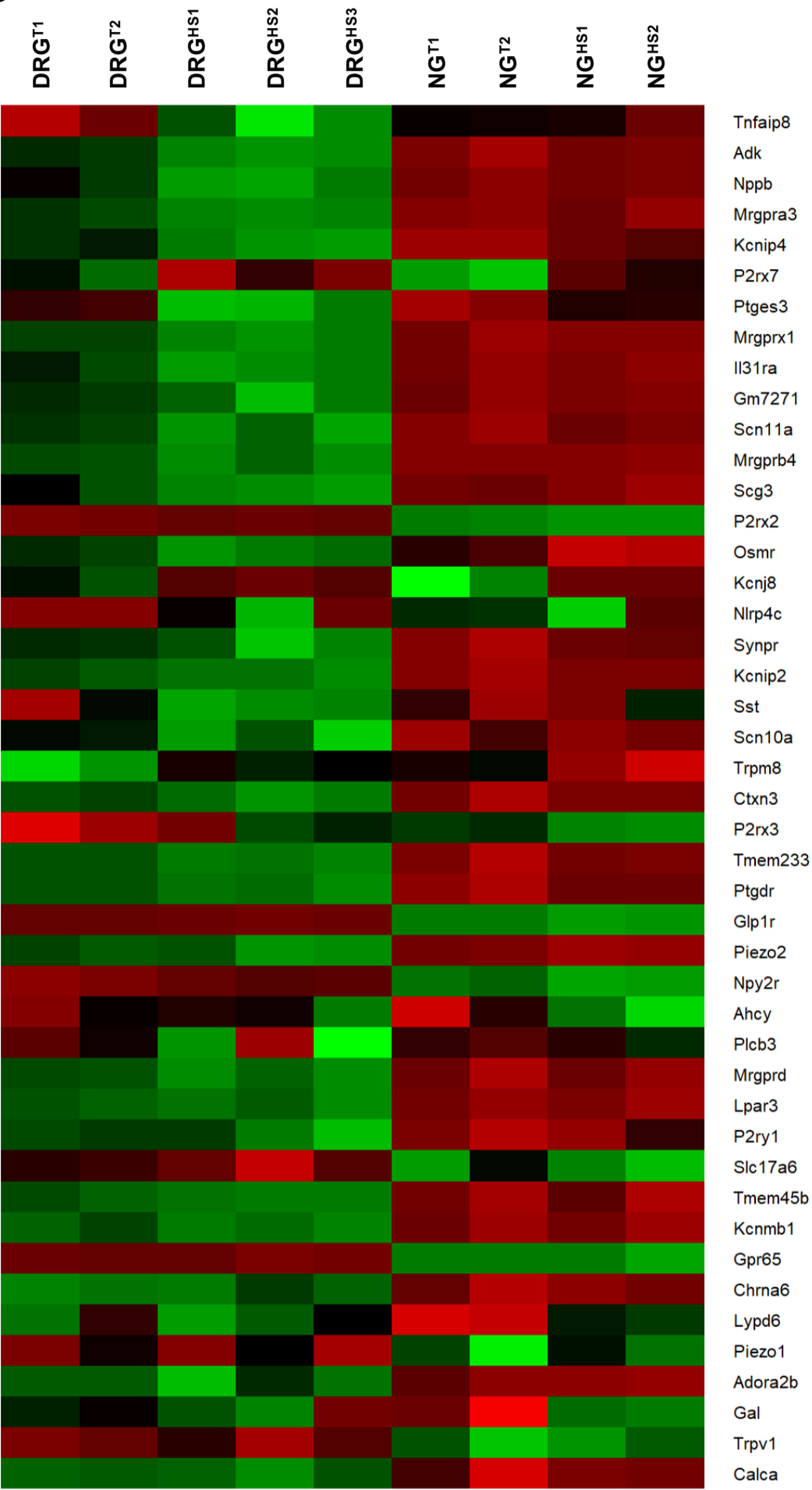

Supplement: Supplementary file 5 — Figure S5: Top 50 up and down genes in heart‐specific and total (A) DRG and (B) NG neurons. (C) The heatmap highlighting the specific genes of interest from Table. [file CPH4-16-e70203-s010.pdf]

Supplementary Figure 6\_R

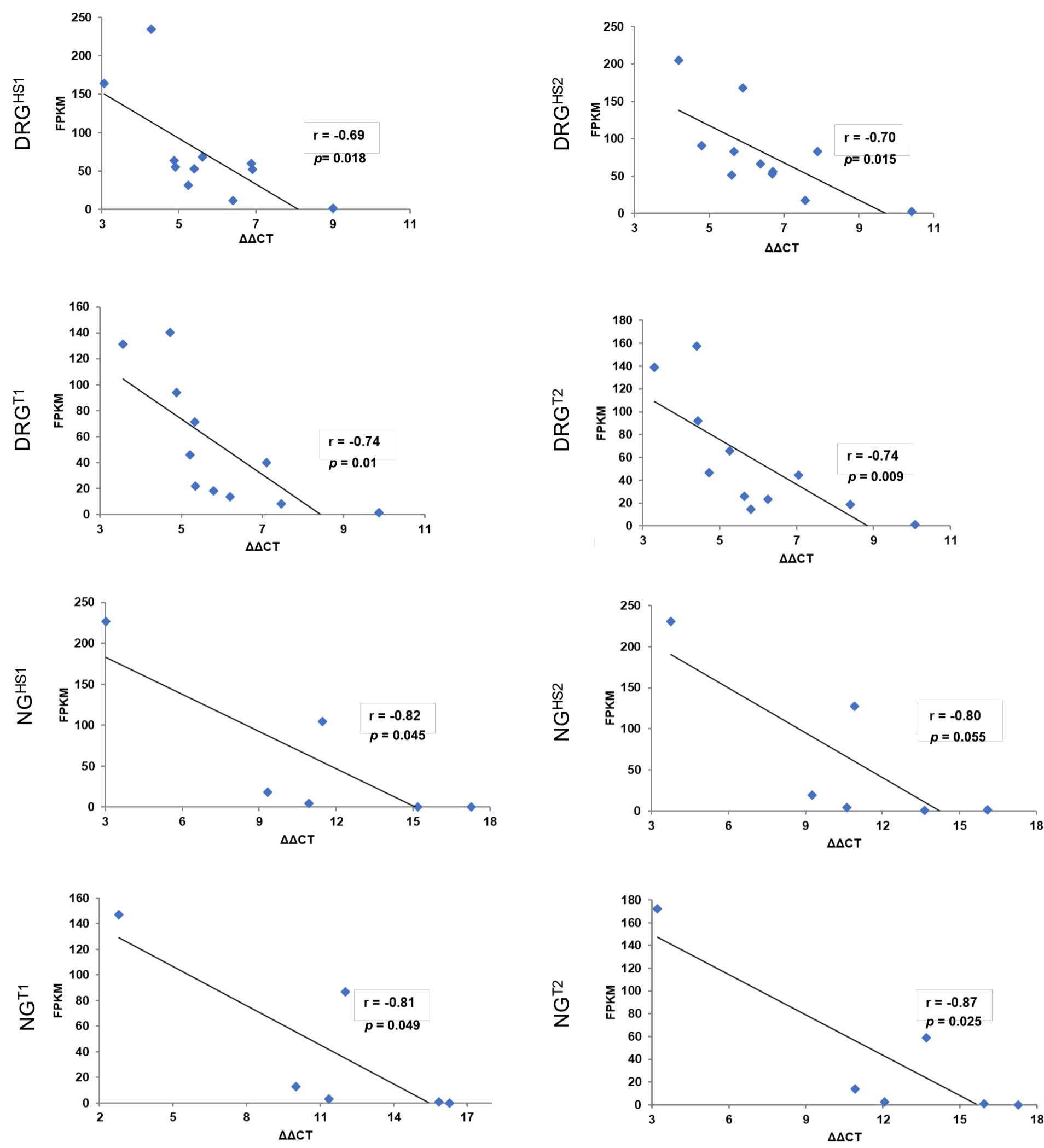

Supplement: Supplementary file 6 — Figure S6: Regression analysis of transcriptome data and qRTPCR analysis for DRG and NG samples. [file CPH4-16-e70203-s008.pdf]

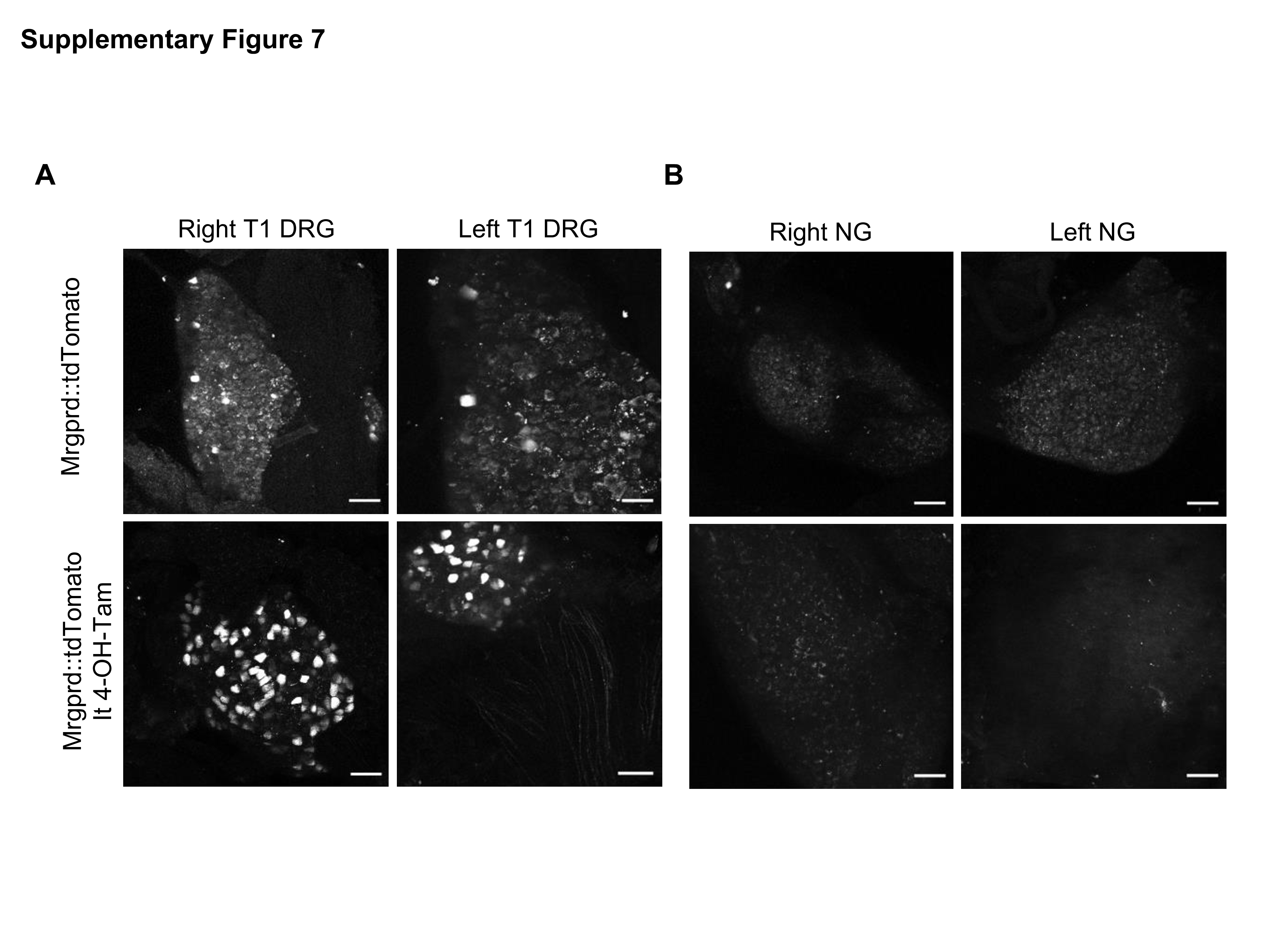

Supplement: Supplementary file 7 — Figure S7: The tdTomato reporter protein imaging reporting Mrgprd expression in the DRG and NG bilateral tissue sections of Mrgprd_cre::tdTomato mice. (A) tdTomato expression before and after tamoxifen application in DRG and (B) NG sections. Scale bars: 50 μm. [file CPH4-16-e70203-s003.jpg]

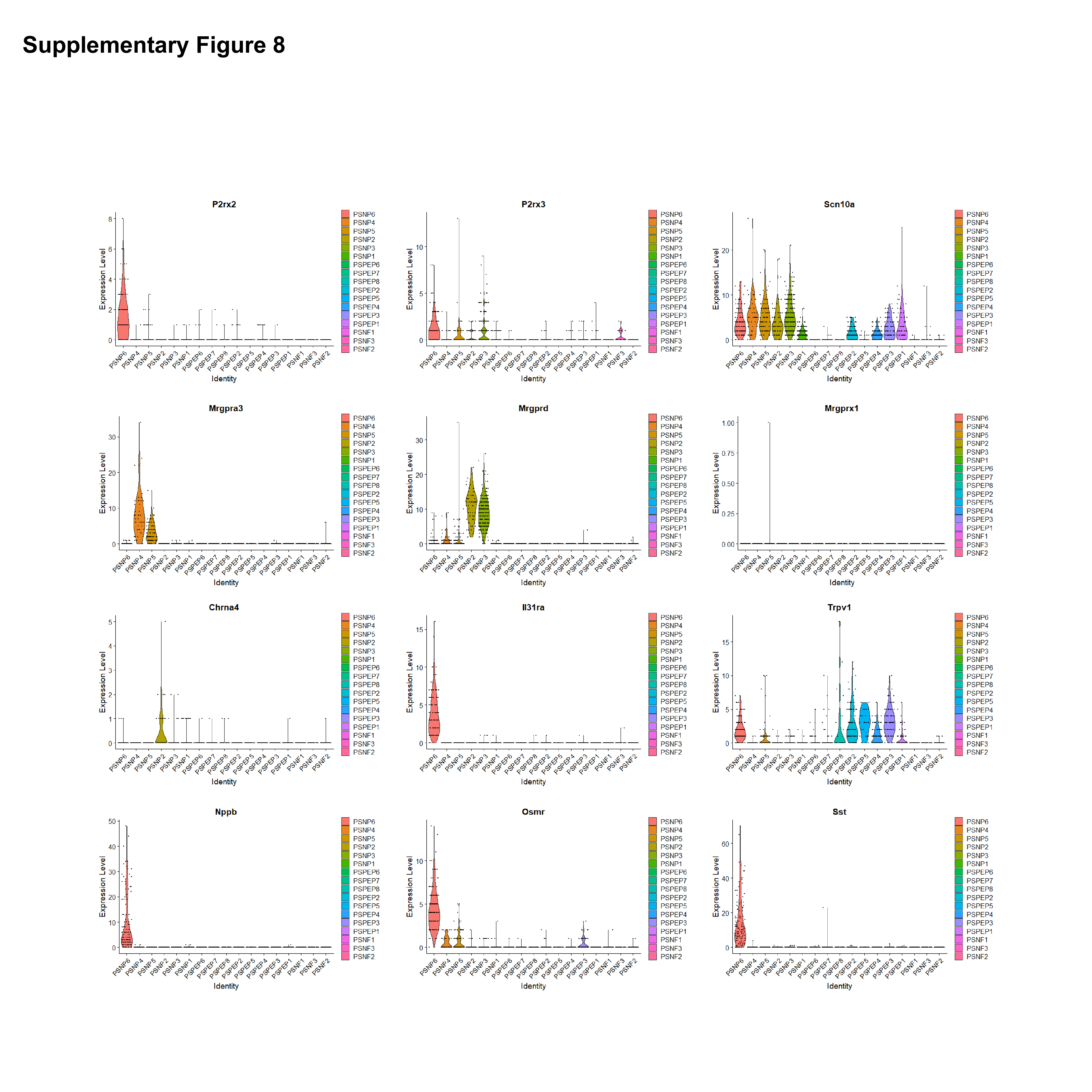

Supplement: Supplementary file 8 — Figure S8: Selected upregulated genes in our DRGHS data exclusively overlapping with a specific spinal sensory neuron cluster out of 17 DRG neuron populations in the single‐cell dataset previously reported in Zeisel et al. (2018). [file CPH4-16-e70203-s009.jpg]

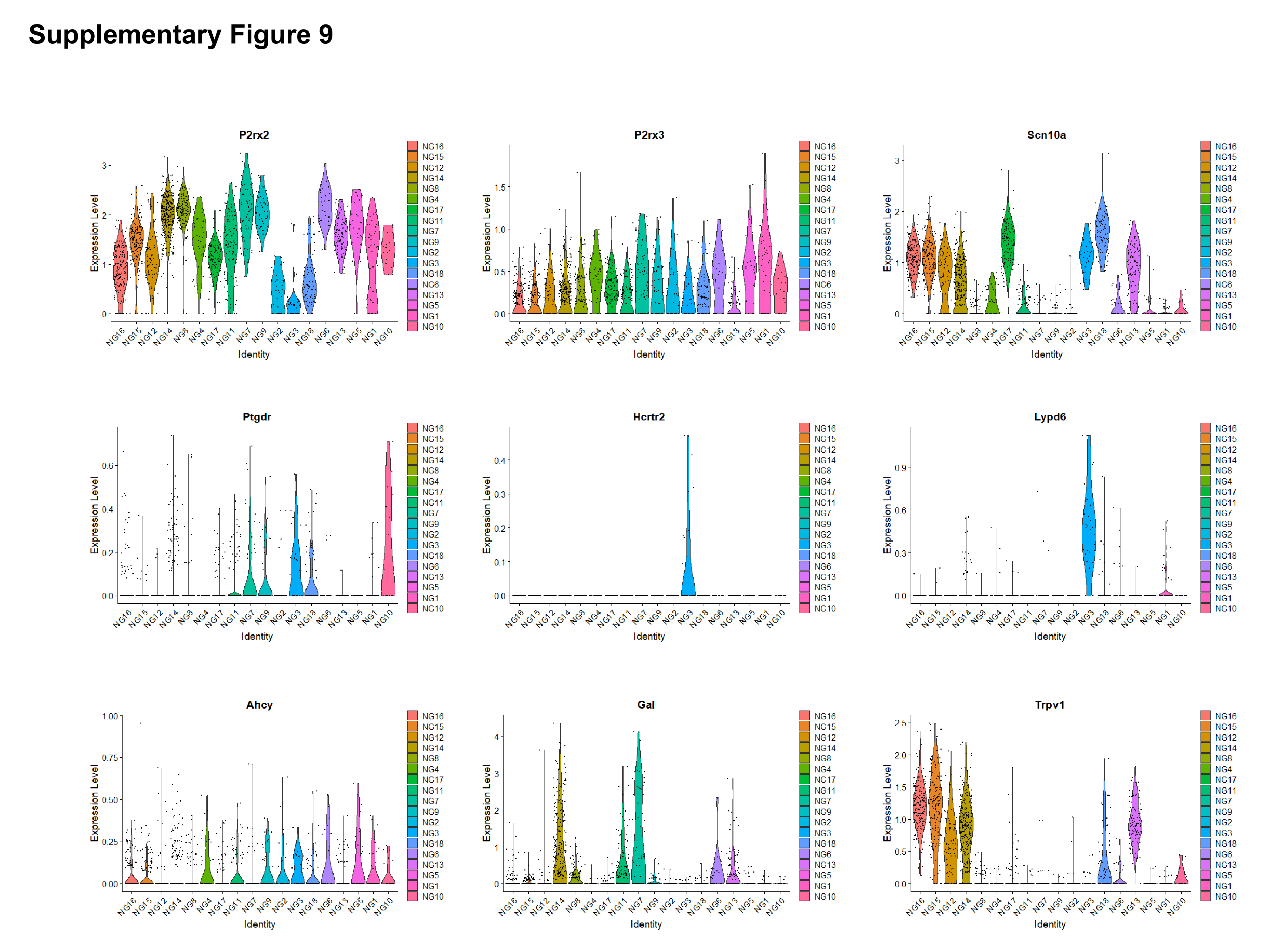

Supplement: Supplementary file 9 — Figure S9: Selected upregulated genes in our NGHS data exclusively overlapping with a specific vagal sensory neuron cluster out of 18 NG neuron populations in the single‐cell dataset previously reported in Kupari et al. (2019). [file CPH4-16-e70203-s002.jpg]
